# Semi-supervised clustering with knowledge-guided representation learning in cryo-electron tomography

**DOI:** 10.1371/journal.pdig.0001619

**Published:** 2026-08-31

**Authors:** Mohamad Kassab, Chengzhi Cao, Vincent Yao, Xiangrui Zeng, Qirong Ho, Min Xu

**Affiliations:** 1 Department of Computer Vision, Mohamed bin Zayed University of Artificial Intelligence, Abu Dhabi, United Arab Emirates; 2 Research Office, Mohamed bin Zayed University of Artificial Intelligence, Abu Dhabi, United Arab Emirates; 3 Department of Radiology, Athinoula A. Martinos Center for Biomedical Imaging, Massachusetts General Hospital, Boston, Massachusetts, United States of America; 4 Department of Radiology, Harvard Medical School, Boston, Massachusetts, United States of America; 5 Department of Machine Learning, Mohamed bin Zayed University of Artificial Intelligence, Abu Dhabi, United Arab Emirates; University of Illinois Urbana-Champaign, UNITED STATES OF AMERICA

## Abstract

The automated discovery of structural patterns in macromolecular complexes remains a central challenge in cryo-electron tomography, particularly in highly heterogeneous datasets. Although fully unsupervised clustering methods have shown promise in grouping subtomograms by structural similarity, they often ignore a crucial source of information: the partial ground truth routinely available to structural biologists from prior studies or manual annotations. In this work, we propose a semi-supervised structural discovery framework that utilizes partial supervision to guide clustering without compromising the ability to uncover previously unknown structures. At the core of our method is a label-anchored probabilistic clustering mechanism that seeds the latent space using a small subset of labeled examples and refines it through a multi-resolution consensus strategy based on PCA-space voting. This is complemented by an entropy-based confidence scoring scheme that attenuates the influence of ambiguous samples, as well as a feature propagation procedure that extends structural labels to low-confidence regions using local similarity in feature space. Together, these components create a stable and adaptive pipeline capable of discovering both known and novel structures. Our approach is efficient, requires as little as 1% of labeled data per class, and consistently produces clearer, more interpretable feature embeddings compared to fully unsupervised methods, with well-separated clusters from the very first iterations. Extensive experiments on simulated and realistic tomographic datasets demonstrate that this semi-supervised strategy significantly improves clustering performance, robustness, and biological relevance in cryo-electron tomography analysis. These methods are integrated as extensions to the existing Deep Iterative Subtomogram Clustering Approach pipeline, enhancing its capability for guided structural discovery.

## Author summary

Cryo-electron tomography (cryo-ET) enables the visualization of macromolecular complexes in 3D inside cells, but each tomogram contains numerous noisy, overlapping structures that are difficult to sort automatically. Existing clustering methods usually treat every subtomogram as completely unknown, while many biologists already have some prior knowledge from earlier studies or manual annotations. In this work, we introduce a semi-supervised clustering framework that can use a very small number of labeled examples—about 1% of subtomograms per known class—to guide the discovery of structural patterns without blocking the appearance of new, previously unseen structures. The method anchors the clustering process with these trusted labels, scores the confidence of each assignment, and propagates labels only where the data support them. Across challenging simulated datasets and real experimental tomograms, our approach produces clearer feature representations and more accurate clusters than a fully unsupervised state-of-the-art method, even under severe noise and limited tilt conditions. This enables more reliable, scalable mining of large cryo-ET collections where most subtomograms remain unlabeled, helping turn growing data resources into biologically meaningful structural insight.

## Introduction

Cryo-ET enables three-dimensional visualization of macromolecular complexes in their native cellular environment while preserving structural integrity [1,2]. By acquiring two-dimensional projection images at multiple tilt angles and reconstructing them into tomograms, cryo-ET preserves both spatial organization and structural integrity without staining or crystallization [1]. Importantly, it maintains *in situ* conditions, minimizing artifacts and retaining the native state of macromolecules [3]. As a result, cryo-ET has become an essential tool in structural cell biology, allowing direct observation of molecular processes and the discovery of previously uncharacterized protein complexes and interactions [4–6].

Despite these capabilities, several fundamental challenges hinder downstream analysis. First, the extremely low signal-to-noise ratio (SNR) in tilt-series, due to low electron doses and crowded cellular environments, degrades structural information [7,8]. Second, the missing wedge artifact, arising from the limited angular range (typically $\pm 60^{\circ }$), produces anisotropic resolution and distortions in 3D reconstructions, complicating tasks such as classification and averaging [9,10]. Finally, traditional particle detection methods such as template matching often rely on downsampled data and low-resolution templates, leading to numerous false positives and poor sensitivity to small or morphologically variable particles [11–13].

Therefore, a major bottleneck lies in the analysis and interpretation of reconstructed tomograms. These volumes contain thousands to tens of thousands of densely packed (e.g., [14,15]), often overlapping macromolecules of various sizes, conformations, and functional states [16,17]. Manual segmentation and identification are prohibitively time-consuming, labor-intensive, and prone to human bias [18], motivating automated and scalable computational approaches.

In cryo-ET, methods are divided into those that recognize known classes and those that discover unknown structures. Most semi-supervised, weakly supervised, and domain adaptation approaches are classification or detection/segmentation pipelines that assume predefined targets and thus do not create new categories, including active learning based classification [19] and semi-supervised autoencoding classification [20]. Detection and segmentation frameworks also focus on recognizing known structures [21–24]. By contrast, discovery-oriented methods operate without labels: DISCA performs high-throughput unsupervised subtomogram clustering for *de novo* discovery [25]; tomoDRGN learns unsupervised latent representations of heterogeneity that can be grouped post hoc [26]; and [27] explore unsupervised routes that ultimately apply k-means to experimental data. Related self- or semi-supervised pattern-mining approaches, such as MiLoPYP, can surface candidate structures with few labels, but their emphasis is on localization and interactive discovery rather than unsupervised clustering into novel structural classes [28].

These considerations motivate our semi-supervised framework. Labels in cryo-ET are costly, variable, and scarce, while large unlabeled datasets are common [3,16,18]. Our goal is to preserve the ability of clustering to reveal a new structure while using limited labels to seed and guide learning. We therefore emphasize stability and accuracy gains from label-guided initialization as the primary benefit and report unknown-class discovery. Adversarial domain adaptation methods [29] have shown that features learned from synthetic labeled data can be transferred to real subtomograms and even recovered structures held-out, but such discovery depends on large synthetic training sets. By contrast, our method achieves iterative discovery directly from the data with minimal labels. To our knowledge, semi-supervision combined with explicit *de novo* subtomogram clustering has received comparatively less attention than classification-oriented semi and weakly supervised pipelines [19–23], while the main discovery engines remain unsupervised clustering and representation modeling [13,25–28,30], which typically assume no prior knowledge and treat all data points as equally unknown [13,25,30–32].

In this work, we introduce a semi-supervised clustering framework for cryo-ET subtomograms that integrates partial ground truth into a modified Gaussian Mixture Model (GMM) with locked labeled assignments. Unlike traditional GMMs that initialize component means randomly or via unsupervised methods, our approach initializes means for known clusters using centroids of labeled features and locks those assignments during EM optimization. To robustly initialize the unlabeled portion before EM, we perform PCA-based majority voting: features are projected into multiple low-dimensional subspaces (e.g., 8, 16, and 32), and a semi-supervised GMM is performed at each resolution. The resulting assignments are aggregated, and each sample receives a pseudo-label by majority vote across projections, providing a stable iteration-0 initialization. We then compute entropy-based confidence scores and propagate labels from high- to low-confidence regions using feature-space similarity. Together, these steps yield a biologically grounded and stable initialization that improves convergence, interpretability, and clustering accuracy in structurally heterogeneous cryo-ET datasets.

**Fig 1:**
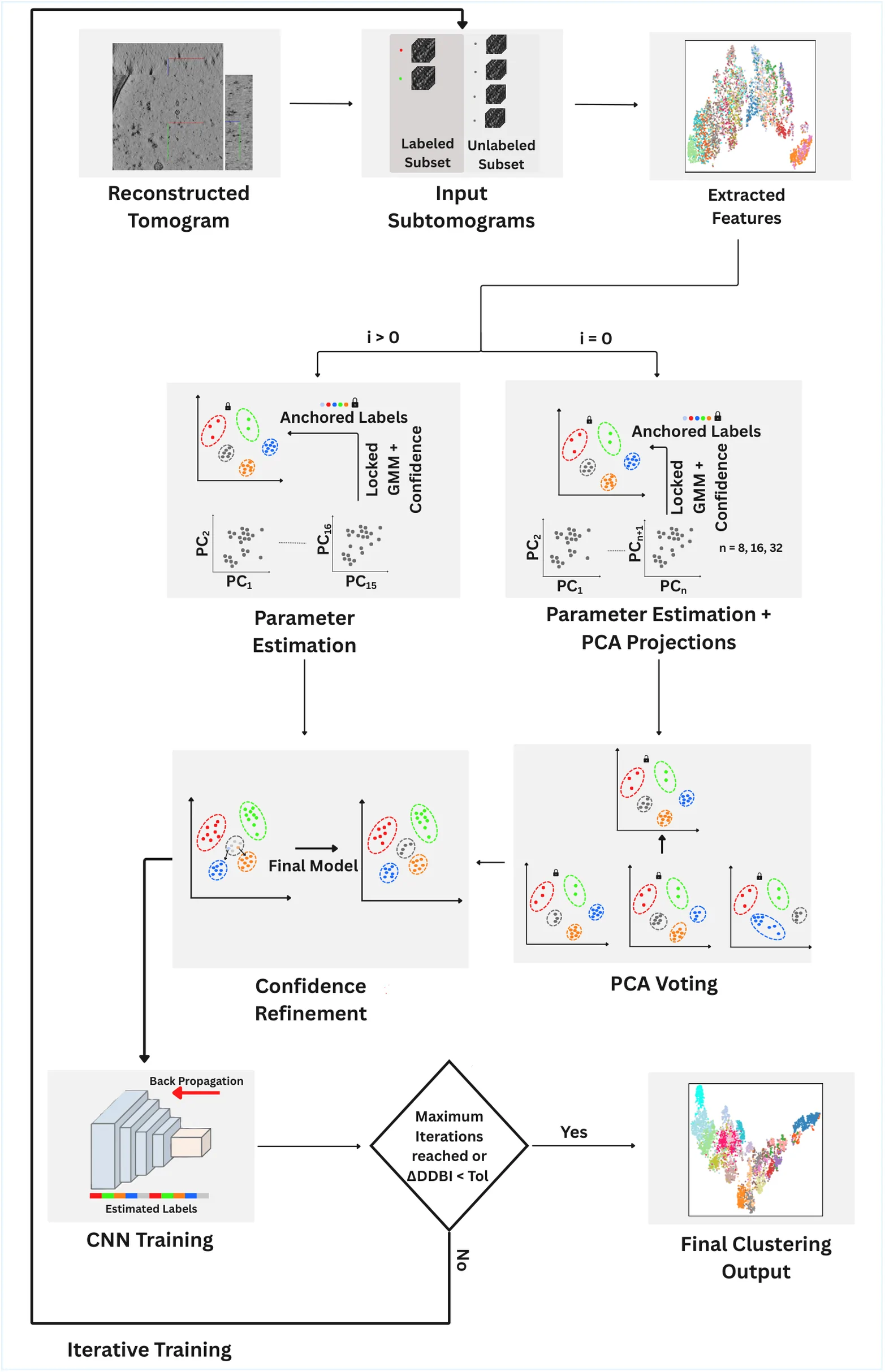
Proposed semi-supervised workflow for subtomogram clustering. The pipeline integrates a small labeled subset with an iterative clustering and training loop. Subtomograms are passed through a CNN for feature extraction, followed by PCA projection and clustering using a GMM with locked responsibilities. PCA voting and BIC are used for model selection. Entropy-based confidence scores and label propagation refine pseudo-labels, which are then used to retrain the CNN. The process continues until convergence.

Fig 1 illustrates the proposed semi-supervised pipeline, which modifies the DISCA model [25]. We begin by extracting subtomograms from reconstructed tomograms, either through the unsupervised Difference of Gaussians (DoG) method [8] or from known coordinates for simulated datasets. Subtomograms are passed through a convolutional neural network (CNN) to obtain high-dimensional features. In the first iteration, the features are projected into PCA spaces with 8, 16, and 32 components; a GMM with locked responsibilities is applied in each space. Parameters (means, covariances) for labeled data are initialized from a small labeled subset (1% per known class) and kept fixed during optimization. For unlabeled data, the GMM estimates cluster assignments, and PCA voting determines consensus labels across projections. The model with the best Bayesian Information Criterion (BIC) is selected for that iteration. Next, entropy-based confidence refinement and label propagation improve pseudo-labels, which are then used to retrain the CNN. The process iterates until convergence or a maximum number of iterations is reached.

## Results

### Performance on simulated datasets using AITom

Table 1 presents the clustering performance on a challenging simulated dataset composed of 23 distinct macromolecular structures, with molecular weights ranging from small complexes like 3QM1 (31.31 kDa) to large assemblies such as 5MRC (3,325.59 kDa) and 4V7R (5,851.65 kDa). Each structure is represented by 400 simulated subtomograms at 32 voxels, totaling 9,200 subtomograms per dataset. Six datasets were generated using AITom [33] under two tilt-angle ranges (±60° and ±40°) and three signal-to-noise ratios (SNR) levels (0.1, 0.03, and 0.01), capturing a highly complex and noise-prone clustering scenario. To evaluate our method’s capability in unknown structure discovery despite its semi-supervised nature, we deliberately sampled only 1% of subtomograms from 18 of the 23 classes, leaving five classes completely unlabeled. This setup enables testing our model’s ability to generalize beyond seen structures while minimizing reliance on labeled data. The original DISCA framework failed to infer the correct number of clusters in most conditions, which limits its ability to report clustering accuracy, since this metric is only valid when the predicted number of clusters matches the ground truth. To enable fair comparison, we evaluated a version of DISCA where the number of clusters is manually fixed to *k* = 23, the true number of ground truth classes. Our semi-supervised approach outperforms both versions of DISCA across all settings, including low-SNR and limited-tilt scenarios. For instance, at SNR 0.03 and ±60° tilt, our method achieves an accuracy of 0.9554 compared to 0.5702 with DISCA (fixed *k* = 23). Even in the most challenging case (SNR 0.01, ± 40°), our method maintains a 0.4113 accuracy, while DISCA drops to 0.2482. This strong performance highlights the robustness of our method, which stems from its stable initialization and semi-supervised design. In every scenario for this dataset, our semi-supervised approach uncovered five unknown classes, indicating novel-structure discovery capability. Unlike unsupervised DISCA, which depends on random initialization and often diverges under noisy or heterogeneous conditions, our method anchors cluster assignments using a small amount of biologically meaningful supervision. Interestingly, under SNR 0.1 with ±40° tilt, DISCA happened to recover the correct number of clusters. However, we interpret this as a result of favorable initialization rather than a systematic advantage, reinforcing the importance of initialization strategies in clustering workflows. Our method addresses this challenge directly, providing consistent and reliable clustering results in a wide range of realistic scenarios.

**Table 1:** Performance results on synthetic datasets simulated via AITom tools (True *k* = 23).

SNR	Simulated $\pm 60^{\circ }$			Simulated $\pm 40^{\circ }$		
	0.10	0.030	0.010	0.10	0.030	0.010
**DISCA**						
k	20	20	20	23	20	20
Homogeneity ↑	0.78	0.54	0.48	0.92	0.81	0.24
Completeness ↑	0.85	0.59	0.57	0.94	0.87	0.25
V-measure ↑	0.82	0.56	0.52	0.93	0.84	0.25
Accuracy ↑	–	–	–	0.85	–	–
**DISCA (fixed *k* = 23)**						
k	23	23	23	23	23	23
Homogeneity ↑	0.91	0.74	0.50	0.92	0.83	0.37
Completeness ↑	0.91	0.78	0.51	0.94	0.87	0.39
V-measure ↑	0.91	0.76	0.51	0.93	0.85	0.38
Accuracy ↑	0.92	0.57	0.35	0.85	0.72	0.25
**Seeded *k*-means (fixed *k* = 23)**						
k	23	23	23	23	23	23
Homogeneity ↑	0.65	0.54	0.12	0.58	0.21	0.09
Completeness ↑	0.66	0.59	0.13	0.59	0.19	0.09
V-measure ↑	0.65	0.56	0.12	0.59	0.20	0.09
Accuracy ↑	0.55	0.49	0.11	0.45	0.19	0.07
**COP-*k*-means (fixed *k* = 23)**						
k	23	23	23	23	23	23
Homogeneity ↑	0.70	0.54	0.21	0.77	0.37	0.16
Completeness ↑	0.73	0.61	0.22	0.78	0.30	0.17
V-measure ↑	0.72	0.57	0.21	0.78	0.33	0.17
Accuracy ↑	0.58	0.32	0.17	0.72	0.39	0.13
**Ours w/ LA-GMM**						
k	23	23	23	23	23	23
Homogeneity ↑	0.96	0.80	0.56	0.91	0.72	0.62
Completeness ↑	0.99	0.82	0.57	0.92	0.74	0.67
V-measure ↑	0.98	0.81	0.57	0.92	0.73	0.65
Accuracy ↑	0.90	0.79	0.54	0.88	0.72	0.47
**Ours w/ LA-GMM + PCA**						
k	23	23	23	23	23	23
Homogeneity ↑	0.97	0.90	0.58	0.91	0.83	**0.63**
Completeness ↑	0.99	0.92	0.58	0.92	0.84	**0.68**
V-measure ↑	0.98	0.91	0.58	0.92	0.84	**0.65**
Accuracy ↑	0.92	0.87	0.56	0.90	0.85	**0.48**
**Ours w/ LA-GMM + PCA + Confidence**						
k	23	23	23	23	23	23
Homogeneity ↑	0.97	0.93	0.58	0.92	0.85	0.62
Completeness ↑	0.98	0.93	0.59	0.93	0.86	0.67
V-measure ↑	0.98	0.93	0.59	0.93	0.86	0.64
Accuracy ↑	0.95	0.93	0.54	0.90	0.87	0.48
**Our Full Model**						
k	23	23	23	23	23	23
Homogeneity ↑	**0.98**	**0.94**	**0.61**	**0.94**	**0.86**	0.52
Completeness ↑	**0.98**	**0.94**	**0.62**	**0.94**	**0.86**	0.56
V-measure ↑	**0.98**	**0.94**	**0.61**	**0.94**	**0.86**	0.54
Accuracy ↑	**0.98**	**0.96**	**0.56**	**0.94**	**0.88**	0.41

We expanded the comparison beyond unsupervised DISCA to determine whether the gains come from partial supervision in general or from the proposed DISCA extension. We added two generic semi-supervised clustering baselines using the same labeled subset: seeded *k*-means, which uses labels only for centroid initialization, and COP-*k*-means, which uses must-link and cannot-link constraints between labeled samples. We also added ablations that introduce the proposed components incrementally: label-anchored GMM (LA-GMM), LA-GMM with PCA-voting initialization, LA-GMM with PCA-voting initialization and confidence filtering, and the full model with label propagation.

The generic semi-supervised baselines did not reproduce the gains of the proposed method. Seeded *k*-means performed poorly under difficult imaging conditions, reaching only 0.07 accuracy at SNR 0.01 and $\pm 40^{\circ }$ tilt. COP-*k*-means improved over seeded *k*-means in some settings but remained below the GMM-based variants. These results indicate that sparse labels alone, when used through standard semi-supervised clustering mechanisms, are insufficient for robust subtomogram clustering.

The ablations show that the improvement comes from the proposed integration of labels into the DISCA framework. LA-GMM substantially improved over seeded and constrained *k*-means, indicating that probabilistic label anchoring is more effective than centroid seeding or pairwise constraints. Compared with DISCA fixed at the true number of clusters, adding label anchoring through LA-GMM substantially improved performance across all six simulated conditions. Adding PCA voting further improved performance, particularly at SNR 0.03, where accuracy increased from 0.79 to 0.87 under $\pm 60^{\circ }$ tilt and from 0.72 to 0.85 under $\pm 40^{\circ }$ tilt. Confidence filtering further improved the SNR 0.03 setting, reaching 0.93 accuracy at $\pm 60^{\circ }$ and 0.88 at $\pm 40^{\circ }$.

The full model achieved the best or tied-best performance in most conditions, with 0.98 accuracy at SNR 0.10 and $\pm 60^{\circ }$ tilt, 0.96 at SNR 0.03 and $\pm 60^{\circ }$ tilt, and 0.94 at SNR 0.10 and $\pm 40^{\circ }$ tilt. The main exception was the most severe condition, SNR 0.01 with $\pm 40^{\circ }$ tilt, where LA-GMM and LA-GMM + PCA achieved higher performance than the full model. This suggests that under extreme noise and missing-wedge artifacts, entropy-based confidence and label propagation can become unreliable. A low-entropy GMM assignment may still be incorrect when the feature space is noise-dominated, and label propagation can amplify these incorrect pseudo-labels by spreading them to neighboring samples.

**Fig 2:**
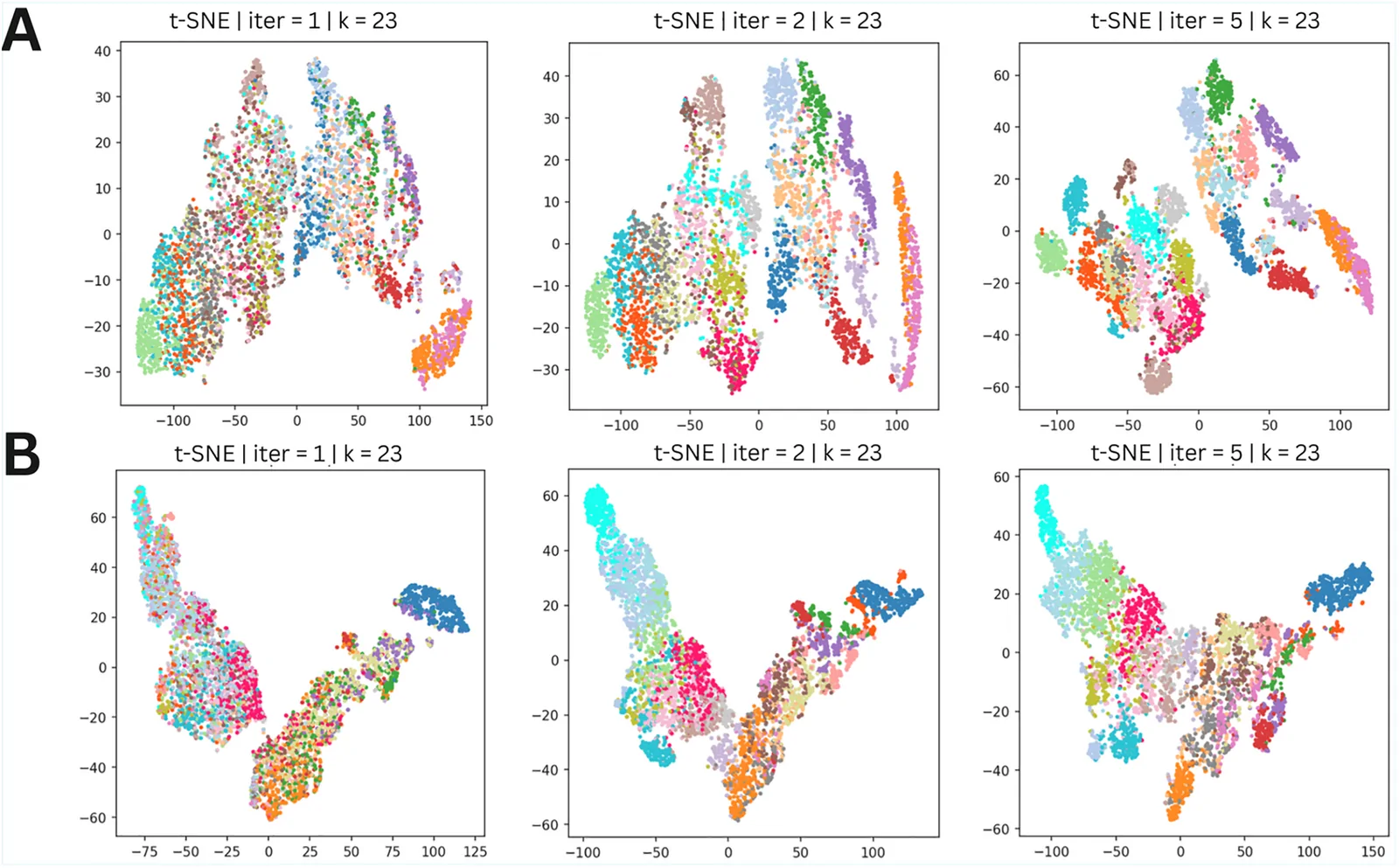
t-SNE visualizations of the reduced feature space for our simulated subtomogram dataset under a ± 60° missing wedge and SNR = 0.03. Panel **A** shows the features learned by our semi-supervised pipeline, where we utilize a small set of locked labels and PCA-voting; this informed starting point yields well-formed islands from the very first iteration and quickly tightens them into clearly separated clusters as training proceeds. Panel **B** illustrates the feature space produced by the fully unsupervised DISCA pipeline with k set to 23 for comparison. At iteration 1, the embedding displays extensive inter-cluster overlap: points from many of the 23 classes occupy the same local neighborhoods, leading to severe color intermixing and poorly defined cluster borders. Although successive training (iteration 5) increases local compactness, the clusters remain densely packed, with minimal inter-cluster gaps and persistent boundary ambiguity. The contrast highlights how our label-aware GMM initialization and confidence-guided refinement provide a superior trajectory in feature space, delivering sharper, more interpretable cluster boundaries throughout optimization.

Fig 2 shows the evolution of the learned feature space for the simulated subtomogram data generated at ±60° tilt and SNR 0.03, visualized with t-SNE, which preserves local neighborhoods and reveals non-linear structure for more interpretable 2-D plots. In Fig 2A, which corresponds to our semi-supervised workflow, the GMM is initialized with a small set of known labels and reinforced by PCA-voting, our method already forms cluster islands by iteration 1, and subsequent iterations further tighten these islands into well-defined and widely separated groups. Fig 2B shows the same iterations for a fully unsupervised DISCA run forced to *k* = 23. Here, the first iteration shows strong overlap among classes, as evidenced by the dense mixing of colors. Although iteration 5 yields slightly more compact groupings, cluster borders remain crowded and ambiguous. The side-by-side comparison shows that our label-aware GMM initialization combined with entropy-guided refinement steers the optimization along a markedly clearer and more discriminative trajectory than the baseline.

### Performance on Dragonfly Cryo-ET dataset

The Dragonfly cryo-ET dataset was constructed from experimental tomograms obtained from the Dryad repository [34]. This dataset is associated with deep-learning training data for cryo-ET segmentation, where multi-slice U-Net models were trained and applied to segment multiple structures within cryo-tomograms. The original tomograms were first manually segmented in Dragonfly to generate supervised annotations for all target classes. These manual annotations were then used to train a U-Net segmentation model. To improve annotation quality and coverage, the segmentation was refined through multiple rounds of U-Net training, automatic tomogram segmentation, manual correction of segmentation errors, and retraining.

After the final segmentation was obtained, binary masks were generated for each annotated structure class. These masks were used to define particle locations and extract subtomograms of size 32^3^ voxels from the original tomograms. The corresponding mask labels were used as ground truth for evaluation. The final Dragonfly subtomogram dataset contained six annotated classes with the following class counts as described in Table 2. We used this dataset to evaluate whether the proposed semi-supervised clustering framework can generalize to experimentally derived subtomograms. To assess unknown-class recovery, we performed a leave-one-class-unlabeled evaluation in which one structural class was withheld completely from the labeled subset, while the remaining classes contributed only 1% labeled examples; this procedure was repeated for each class so that every class served once as the fully unlabeled unknown class.

**Table 2:** Structural discovery on the experimental Dragonfly cryo-ET dataset.

Fully unlabeled class	Number of subtomograms	Recovery accuracy ↑
Membrane	1,294	0.65
Actin	4,641	0.23
Cofilactin	1,042	0.31
Microtubule	318	0.61
Ribosome	484	0.74
TriC	253	0.52

Table 2 shows that our model achieved the strongest recovery for Ribosome, Membrane, and Microtubule, with accuracies of 0.74, 0.65, and 0.61, respectively. TriC was recovered with moderate accuracy of 0.52, whereas Actin and Cofilactin showed lower recovery, with accuracies of 0.23 and 0.31.

The lower performance for Actin and Cofilactin likely reflects their structural similarity. Both are filament-associated classes, and Cofilactin is closely related to actin-containing filament structures. When one of these classes is completely withheld from supervision, the model may assign its subtomograms to the other filament-like class or split them across related clusters, reducing aligned accuracy. This suggests that unknown-class recovery in experimental cryo-ET data is more difficult when the withheld class is morphologically similar to a labeled class. The moderate performance for TriC may instead reflect its smaller and more compact structural appearance, which can make its subtomograms less distinctive in the learned feature space than larger or more spatially extended structures. Overall, these results show that the method can recover several fully unseen structures in real cryo-ET data, but recovery is affected by structural distinctiveness, morphological similarity, and object size.

### Performance on CZII dataset

**Fig 3:**
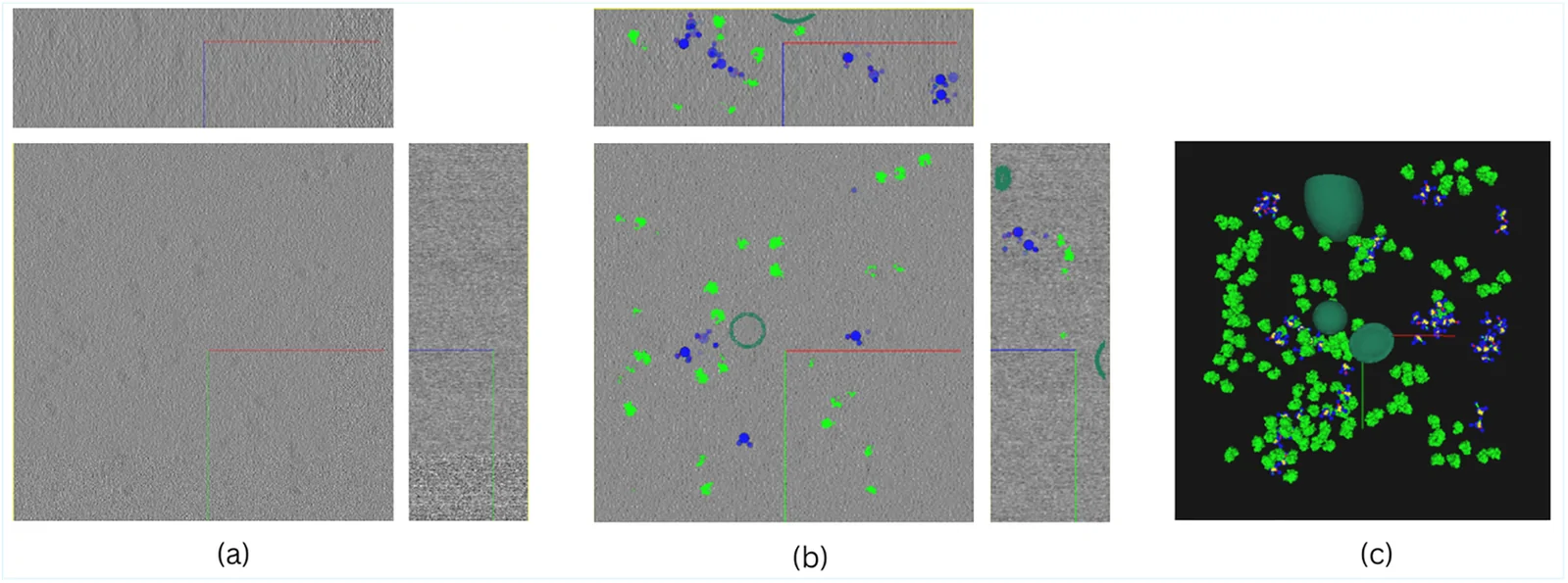
Representative example from the CZII Cryo-ET Object Identification Challenge dataset. **(a)** Raw tomogram slice. **(b)** The same slice with segmentation overlays, highlighting annotated macromolecular classes (membrane, cytosolic ribosomes, and beta-galactosidase). **(c)** 3D rendering of the segmented subtomograms that were extracted to build the dataset.

Table 3 presents the clustering performance of different methods evaluated on a subset of the cryo-ET Object Identification Challenge dataset [35] obtained from CZII (Chan Zuckerberg Institute for Advanced Biological Imaging). As shown in Table 3, the unsupervised DISCA model failed to infer the correct number of clusters, reporting five clusters instead of the expected three. Consequently, clustering accuracy could not be reported in this case. To enable a fair comparison, we evaluated DISCA again with the number of clusters fixed to *k* = 3, which yielded an accuracy of 0.5117—still notably lower than our semi-supervised models. When applying our semi-supervised framework without PCA voting or confidence refinement, performance improved across all metrics, particularly accuracy (0.7676) and completeness (0.4700), highlighting the benefit of incorporating partial supervision even without advanced refinement strategies. Finally, the full version of our model, which includes both PCA voting and confidence refinement, achieved the highest scores in all metrics, with an accuracy of 0.8329, homogeneity of 0.5594, completeness of 0.5788, and V-measure of 0.5689. This substantial improvement demonstrates the effectiveness of our proposed refinements and confirms their role in boosting clustering performance in complex, real-world cryo-ET datasets (Fig 3).

**Table 3:** Performance on processed datasets from CZII - CryoET Object Identification Challenge (True *k* = 3).

Method	*k*	Homogeneity ↑	Completeness ↑	V-measure ↑	Accuracy ↑
DISCA	5	0.32	0.33	0.32	–
DISCA (fixed *k* = 3)	3	0.38	0.48	0.43	0.51
Ours w/o confidence & PCA voting	3	0.44	0.47	0.45	0.77
Ours with confidence & PCA voting	3	**0.56**	**0.58**	**0.57**	**0.83**

### Performance on simulated datasets using PolNet

**Fig 4:**
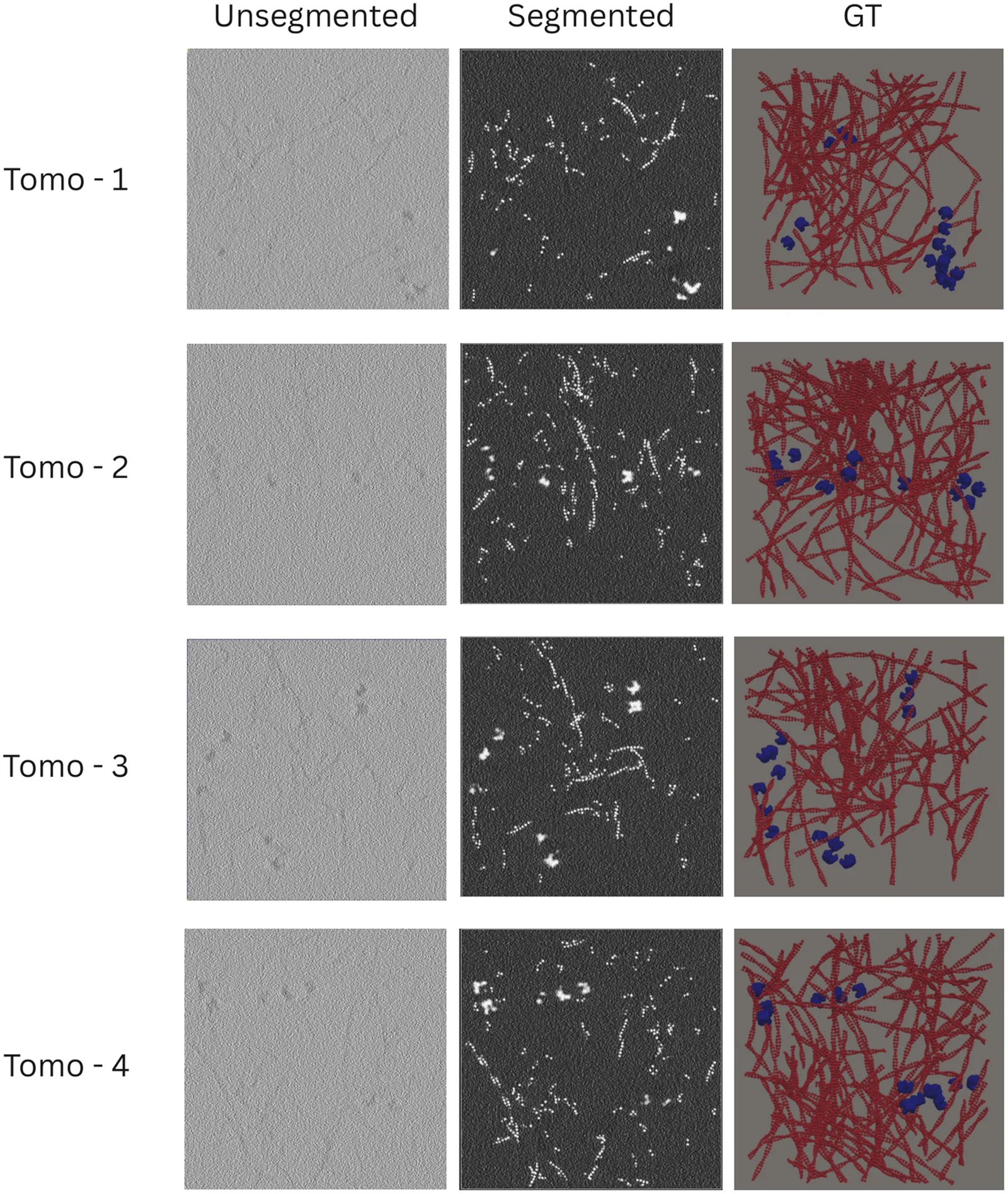
Visualization of the four simulated tomograms generated with PolNet. For each tomogram, the first column shows a slice of the raw tomogram at SNR = 0.1, the second column displays the semi-supervised clustering results embedded on the same slice, and the third column presents the complete 3D ground truth with actin filaments in red and 1BXN macromolecules in blue.

Table 4 reports the performance of our method when evaluated on tomograms generated using PolNet. PolNet is an open-source Python framework for synthesizing realistic cryo-electron tomograms by combining parametric geometric models with stochastic spatial organization to recreate low-order cellular structures such as membranes, filament networks, and macromolecular complexes [36]. The framework also supports the incorporation of realistic imaging artifacts, including Gaussian noise, micrograph misalignment, and the missing wedge, enabling the creation of synthetic datasets that closely emulate experimental cryo-ET. For this experiment, we configured PolNet to generate four tomograms containing mixtures of actin filaments and the macromolecule 1BXN, acquired at a challenging low SNR of 0.1, with tilt-series parameters consistent with standard cryo-ET acquisition. Our semi-supervised clustering model achieved consistently high scores across all metrics, with homogeneity, completeness, V-measure, and accuracy values exceeding 0.98 for every tomogram. Fig 4 presents the four tomograms generated using PolNet and visualized with its built-in rendering tools. The first column displays a representative slice from the raw tomogram, illustrating the low signal-to-noise ratio conditions. The second column overlays the clustering results onto the same tomogram slice, highlighting the detected structures in their spatial context. The third column shows the complete 3D structural ground truth for each tomogram, with actin filaments represented in red and 1BXN macromolecules in blue.

**Table 4:** Performance of the proposed semi-supervised method on four tomograms generated using PolNet (True *k* = 2).

Tomogram	*k*	Homogeneity ↑	Completeness ↑	V-measure ↑	Accuracy ↑
Tomo-1	2	0.98	0.99	0.99	0.99
Tomo-2	2	0.98	0.98	0.98	0.98
Tomo-3	2	0.99	0.98	0.990	0.99
Tomo-4	2	0.98	0.99	0.988	0.98

### Sensitivity analysis

To evaluate the robustness of the proposed semi-supervised framework, we performed sensitivity analyses for three key choices: the proportion of labeled subtomograms, the number of neighbors used during confidence-based label propagation, and the entropy-confidence threshold used to define high-confidence samples. All sensitivity analyses were performed on a challenging 23-class simulated subtomogram dataset generated at SNR = 0.001 with a $\pm 40^{\circ }$ missing-wedge tilt range. This setting was selected to approximate the severe noise and limited angular sampling encountered in experimental cryo-ET data.

#### Sensitivity to labeled subset proportion.

**Fig 5:**
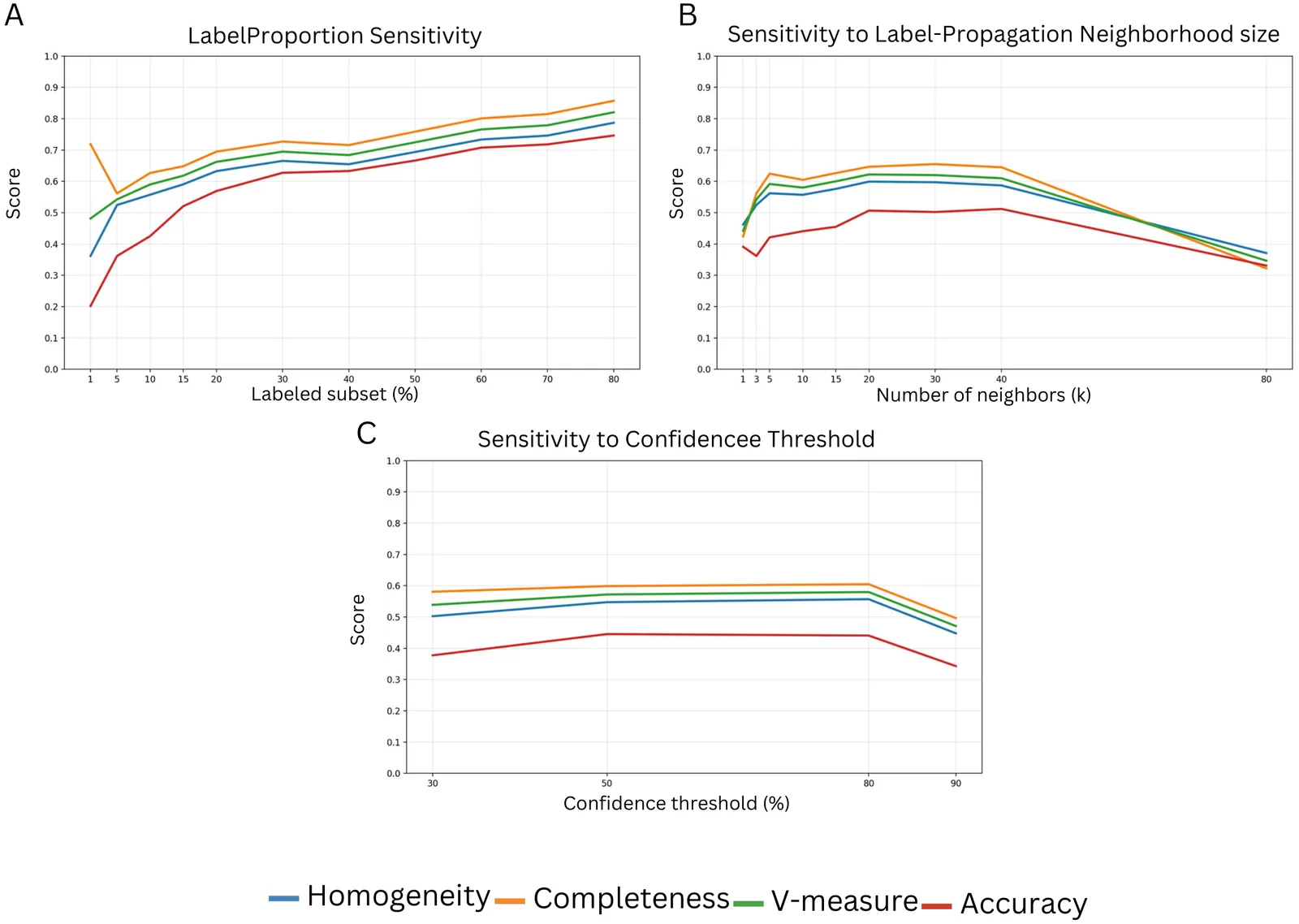
Sensitivity analysis under extreme low-SNR simulated conditions. Sensitivity analyses were performed on a 23-class simulated subtomogram dataset generated at SNR = 0.001 with a $\pm 40^{\circ }$ missing-wedge tilt range. **(A)** Effect of increasing the labeled subset from 1% to 80%. Performance improves with additional labels, supporting the use of 1% as a conservative low-supervision setting rather than an optimal label fraction. **(B)** Effect of the number of high-confidence neighbors used in confidence-based label propagation. Moderate neighborhood sizes improve performance, whereas very large neighborhoods lead to oversmoothing. **(C)** Effect of the entropy-confidence threshold used to define high-confidence samples. A threshold of 0.80 provides the best V-measure, while 0.90 is overly restrictive. Blue, orange, green, and red curves denote homogeneity, completeness, V-measure, and accuracy, respectively.

Fig 5A shows the effect of increasing the labeled subset from 1% to 80%. As expected, performance improved as more labeled subtomograms were provided. Accuracy increased from 0.20 at 1% labeled data to 0.75 at 80%, while V-measure increased from 0.48 to 0.82. Homogeneity also increased substantially, from 0.36 to 0.79, indicating that additional labels helped produce purer clusters. This confirms that the model benefits from stronger supervision. However, the purpose of using 1% in the main experiments was not to claim that 1% is optimal, but to evaluate the method under a deliberately strict low-annotation setting. Thus, the 1% condition serves as a conservative test of whether the framework can operate when expert annotations are scarce.

#### Sensitivity to the number of neighbors in label propagation.

Fig 5B evaluates the number of nearest high-confidence neighbors used during label propagation, testing *k*={1,3,5,10,15,20,30,40,80}. Using only one neighbor gave the weakest performance, with V-measure of 0.44 and accuracy of 0.39, indicating that single-neighbor propagation is sensitive to local noise. Performance improved when multiple high-confidence neighbors were used, reaching the best V-measure at *k* = 20 and the best accuracy at *k* = 40. However, performance dropped sharply at *k* = 80, where V-measure decreased to 0.35 and accuracy to 0.33. This suggests that moderate neighborhood sizes improve robustness, whereas excessively large neighborhoods oversmooth labels across nearby clusters. Our main experiments used *k* = 5 as a conservative local-neighborhood setting, while the sensitivity analysis showed that larger moderate neighborhoods can further improve performance under extremely low-SNR conditions.

#### Sensitivity to confidence threshold.

Fig 5C shows the effect of varying the entropy-confidence threshold used to define high-confidence samples for label propagation. We tested thresholds of 0.30, 0.50, 0.80, and 0.90. Performance improved from threshold 0.30 to 0.80, with V-measure increasing from 0.54 to 0.58. However, increasing the threshold to 0.90 reduced performance, with V-measure decreasing to 0.47 and accuracy to 0.34. This indicates that too low a threshold may allow unreliable pseudo-labels to propagate, whereas too high a threshold can leave too few samples available for effective refinement. The threshold of 0.80 provided the best V-measure and was therefore used as the default setting.

Overall, these sensitivity analyses show that the proposed framework benefits from additional labeled data, but also remains evaluable under a stringent 1% supervision regime. They also show that confidence-based label propagation is most effective when it uses a moderately sized local neighborhood and a conservative, but not overly restrictive, confidence threshold.

## Discussion

The results across the different datasets demonstrate that integrating partial supervision into the clustering process yields substantial and consistent improvements over fully unsupervised methods. By anchoring cluster initialization with biologically meaningful labeled examples and reinforcing this initialization through multi-resolution PCA voting, our framework achieves greater stability and robustness in both high- and low-SNR regimes. This addresses a key limitation of unsupervised approaches, which often struggle with random initialization and the instability it induces under noisy or heterogeneous conditions. In addition, unlike conventional semi-supervised methods, ours maintains unsupervised-level discovery of unknown structures.

In simulated datasets with 23 macromolecular classes, our method consistently outperformed DISCA in homogeneity, completeness, V-measure, and precision, even in challenging $\pm 40^{\circ }$ tilt and SNR 0.01 scenarios. The performance gap was particularly pronounced when the unsupervised baseline failed to recover the true number of clusters, underscoring the value of stable, knowledge-guided initialization. The t-SNE visualizations further highlight this advantage, showing that our label-aware GMM initialization produces well-formed and clearly separated clusters from the earliest iterations, in contrast to the diffuse and overlapping clusters observed in the unsupervised baseline. Furthermore, the comparisons in Table 1 further clarify that the improvement is not merely a consequence of adding a small number of labeled examples. Generic semi-supervised baselines, including seeded *k*-means and COP-*k*-means, did not reproduce the gains of the proposed method, particularly under low-SNR and limited-tilt conditions. In contrast, the ablation sequence showed that label-anchored GMM, PCA voting, confidence filtering, and label propagation contribute incrementally to performance. This supports the interpretation that the main benefit arises from how sparse labels are integrated into the DISCA framework, rather than from partial supervision alone.

Evaluation on the CryoET Object Identification Challenge dataset confirmed the generalizability of our approach to real experimental data. Even with only 1% of the samples labeled, the semi-supervised model improved accuracy from 0.5117 (DISCA, fixed *k* = 3) to 0.8329 when both PCA voting and confidence-based refinement were employed. The performance gain from adding the refinement stage demonstrates its ability to correct uncertain assignments and sharpen decision boundaries, further enhancing the biological interpretability of the clusters.

The Dragonfly leave-one-class-unlabeled experiment further demonstrates that the framework can recover fully unseen structures in experimental cryo-ET data. Recovery was strongest for Ribosome, Membrane, and Microtubule, while Actin and Cofilactin were more difficult to separate, likely because of their related filamentous morphology. This result highlights an important practical limitation: unknown-class discovery is feasible in real tomograms, but recovery depends on structural distinctiveness and feature-space separability. Thus, the method is most effective when the withheld structure forms a sufficiently distinguishable cluster, and less reliable when the unknown class is morphologically close to a labeled class.

The PolNet experiments provide additional evidence of the method’s robustness, achieving near-perfect scores across all metrics for four distinct tomograms. These results indicate that the proposed framework is not only effective in noisy and heterogeneous conditions but also adaptable to a variety of structural contexts and imaging conditions.

The sensitivity analyses also help contextualize the choice of hyperparameters. Increasing the labeled subset from 1% to 80% improved performance, as expected, confirming that additional supervision strengthens cluster anchoring. However, the 1% setting remains valuable as a deliberately stringent low-annotation regime, rather than an optimal label fraction. The neighborhood-size and confidence-threshold analyses show that confidence-based propagation works best with a moderately sized local neighborhood and a conservative, but not overly restrictive, confidence threshold. These results indicate that the method is robust over a reasonable parameter range, while also showing that overly broad propagation or overly strict confidence filtering can reduce performance under extremely low-SNR conditions.

While these results confirm the strength of our framework, they also highlight the broader context of cryo-ET research. The CryoET Data Portal [37] hosts more than 19,000 tomograms in 327 datasets (as of 2025). Of these, only about 124 tomograms include annotations or partial annotations, while the majority remain unlabeled. EMPIAR [38] contains 2,389 entries (as of 2025), spanning cryo-EM images, cryo-ET, and related modalities such as X-ray tomography. However, only a small subset of entries is manually annotated and routinely reused for machine learning applications [39]. This scarcity of labeled data has become a bottleneck for supervised training, underscoring the need for semi-supervised approaches that exploit a small labeled subset without imposing the full burden of exhaustive annotation while leveraging the large reservoir of unlabeled data.

In real-world medical applications, domain experts often possess limited but valuable ground truths [40]; in cryo-ET, this includes previously identified structures or annotated subtomograms that can guide clustering. Incorporating such partial knowledge can enhance biological interpretability and robustness, especially in datasets with high structural diversity and imaging noise [19,20,41]. Readily available GUI tools support expert-driven annotation for semi-supervised cryo-ET workflows [34,42–49]. As our experiments show, clustering stability and accuracy improve when utilizing such expert knowledge without sacrificing capacity for novel discovery.

Overall, this work establishes that incorporating partial supervision into cryo-ET clustering pipelines can significantly enhance structural discovery without compromising the ability to identify novel structures. The combination of label-guided GMM initialization, PCA-based consensus, and confidence-driven refinement forms a principled approach to bridge supervised and unsupervised paradigms in subtomogram analysis.

## Materials and methods

### Datasets

#### Data simulation using AITom tools.

To simulate realistic subtomograms, we selected 23 protein structures from the Protein Data Bank (PDB) [50], covering a broad range of molecular weights (from 31.31 kDa to 5,851.65 kDa). The selected entries include:

- Small to mid-size proteins: 1BXN (279.81 kDa), 1QVR (296.68 kDa), 1S3X (42.75 kDa), 1U6G (238.82 kDa), 2CG9 (188.73 kDa), 3CF3 (270.87 kDa), 3D2F (236.11 kDa), 3GL1 (84.61 kDa), 3H84 (79.04 kDa), 3QM1 (31.31 kDa).
- Large complexes: 2UV8 (1,310.37 kDa), 3IPM (533.09 kDa), 3J9I (659.00 kDa), 4CR2 (1,309.28 kDa), 4V4R (2,277.81 kDa), 4V7R (5,851.65 kDa), 4V94 (1,952.74 kDa), 5GJV (413.26 kDa), 5MRC (3,325.59 kDa), 6RD4 (881.10 kDa), 6UTJ (838.60 kDa).
- Ribosomal subunits extracted from 4V4R: 30S and 50S subunits.

**Fig 6:**
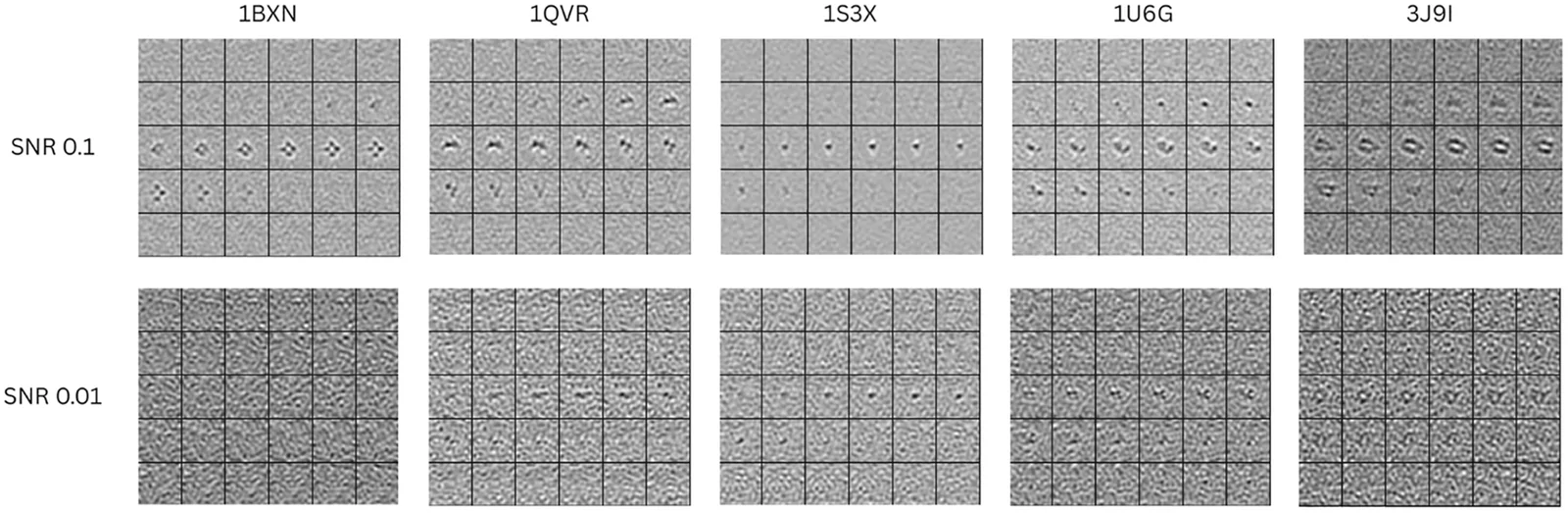
AITom subtomogram simulation. Sample of subtomogram complexes generated using the AITom generator and projected onto a 2D plane for visualization. Each column represents a different PDB structure under two SNR conditions.

Each PDB structure was first converted into a 3D density map using Situs 3.2 [51]. The maps were resized and formatted into volumes with 10 Å voxel spacing and 40 Å target resolution. A sample of the generated subtomograms is shown in Fig 6.

To generate realistic subtomograms, we followed a three-stage simulation process:

1. ***Random Transformations:*** Each density volume was randomly rotated and shifted in 3D space to simulate structural heterogeneity and positional variation.
2. ***Cryo-ET Forward Simulation:*** Transformed volumes were passed through a model that mimics physical acquisition in cryo-ET. The simulation included:
  - Applying a missing wedge in Fourier space to simulate tilt limitations.
  - Modulation of the signal with a contrast transfer function (CTF), parameterized by a defocus value of −5 μm, spherical aberration of 2.7 mm, accelerating voltage of 300 keV, and pixel size of 1 nm.
  - Adding Gaussian noise to reach a specified SNR, followed by frequency-domain bandpass filtering to enhance interpretability.
3. ***Dataset Generation:*** For each protein structure, 400 subtomograms were simulated under six different SNR conditions, resulting in six distinct datasets and a total of 55,200 subtomograms. To enable semi-supervised learning, 1% of subtomograms from each class were designated labeled, while the remaining samples were treated as unlabeled for downstream analysis.

#### Data Simulation with PolNet.

**Fig 7:**
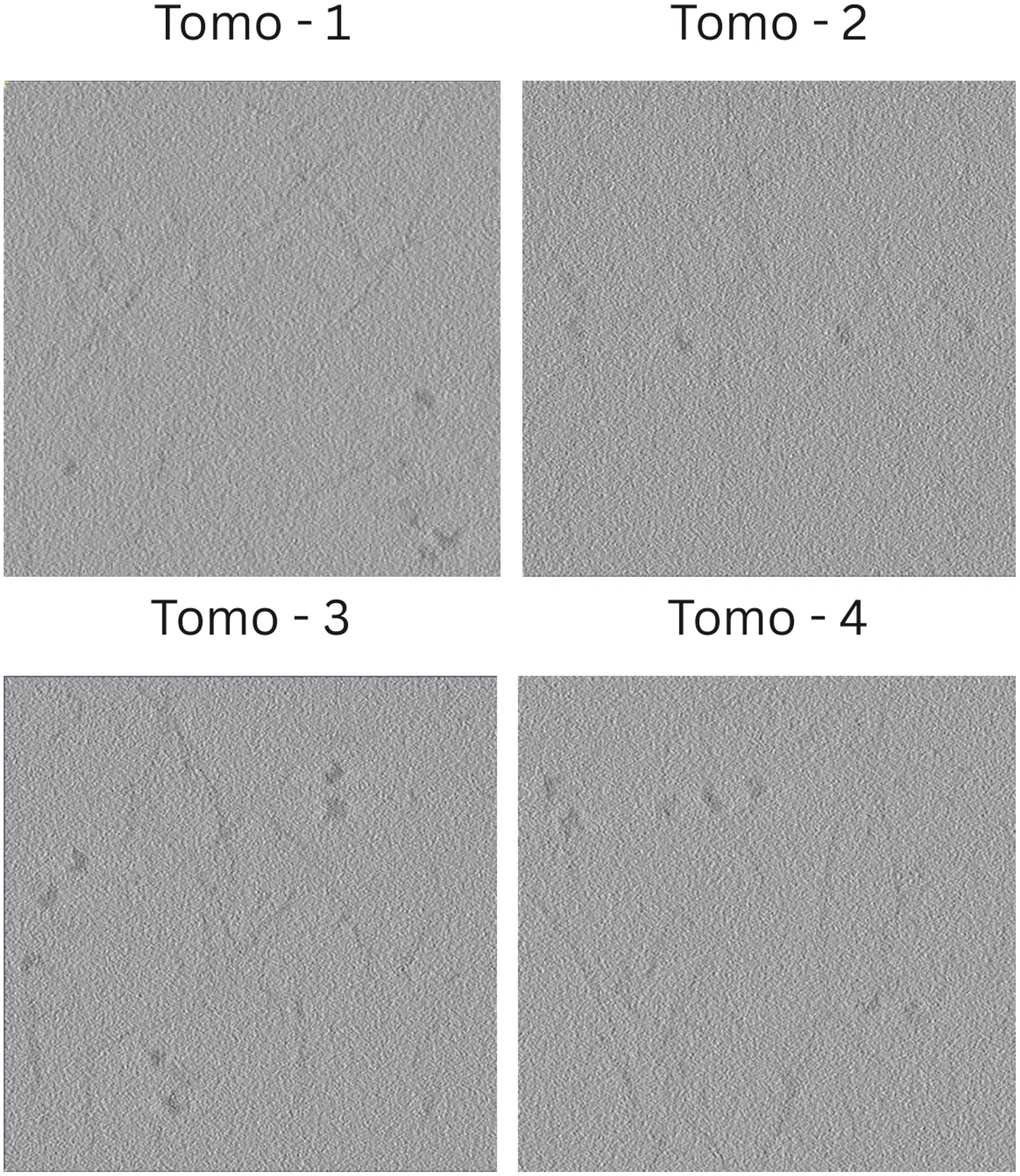
PolNet tomogram simulation. Each panel show a single slice of the tomograms generated using the PolNet.

PolNet employs parametric and stochastic models to generate geometries and spatial organizations, followed by a trial-and-error insertion algorithm to achieve biologically plausible crowded environments [36]. It can also introduce imaging distortions such as Gaussian noise, micrograph misalignment, and missing wedge effects to mimic experimental cryo-ET conditions [36]. We configured PolNet to generate four tomograms as shown in Fig 7 with a volume size of 400×400×100 voxels and an effective volume defined by offsets of 4–396 in X and Y and 4–96 in Z. The voxel size was set to 10 Å, with a maximum of 20 tries for monomer placement and 100 tries for polymer placement. For actin filaments, the decimation factor for surface representation was 0.9, and micrograph misalignment was modeled with a mean of 1 pixel, maximum of 1.5 pixels, and sigma of 0.2 pixels. The simulated micrographs had an SNR of 0.1, and tilt series were acquired from $-60^{\circ }$ to $61^{\circ }$ in $3^{\circ }$ increments. Each tomogram contained a mixture of actin filaments and the macromolecule 1BXN.

#### Experimental tomograms - CZII CryoET object identification challenge.

This dataset is obtained from CZII (Chan Zuckerberg Institute for Advanced Biological Imaging) [35] and it comprises tomograms acquired using a Krios G4 equipped with a Falcon 4i detector and SelectrisX energy filter. From this dataset, we selected three *in-vitro* macromolecular structures with consistent annotations: membrane, cytosolic ribosomes, and beta-galactosidase, which were extracted across multiple tomograms and processed into subtomograms of size 40^3^ voxels (shown in Fig 3A). Each subtomogram was denoised and low-pass filtered to mitigate reconstruction artifacts. The resulting dataset consists of 1,239 subtomograms balanced across the three classes. To simulate a realistic semi-supervised setting and test the model’s ability to discover unknown structures, only 1% of the subtomograms (from the three selected classes) were randomly selected as labeled, the remaining samples left unlabeled.

### Experimental Dragonfly Cryo-ET dataset

The experimental Dragonfly cryo-ET dataset was generated from tomograms obtained from the Dryad repository associated with deep learning-based cryo-ET segmentation [34]. The tomograms were first manually annotated in Dragonfly to create supervised labels for all target structural classes. These annotations were used to train a U-Net model, which was then applied to segment additional tomogram regions; the predictions were manually inspected, corrected, and used for further rounds of training. After this iterative annotation, prediction, correction, and retraining process, class-specific masks were generated and used to define subtomogram coordinates and class labels. Subtomograms of size 32^3^ voxels were extracted from the original tomograms, producing six annotated classes: Actin (*n* = 4,641), Cofilactin (*n* = 1,042), Membrane (*n* = 1,294), Microtubule (*n* = 318), Ribosome (*n* = 484), and TriC (*n* = 253). To evaluate unknown-structure discovery, we used a leave-one-class-unlabeled protocol in which one class was completely withheld from the labeled subset while the remaining classes contributed only 1% labeled examples.

### Methods

#### Training details.

All models were trained and tested on a high-performance GPU cluster equipped with a single NVIDIA A100 GPU (40 GB memory) and 64 GB of system RAM. We trained with Adam (lr = 1x10^-4^), batch size = 64, label smoothing = 0.2, and GMM covariance regularization = 1x10^-5^.

#### DISCA method.

The Deep Iterative Subtomogram Clustering Approach (DISCA) [25] is an unsupervised framework that automatically sorts heterogeneous subtomograms into homogeneous structural subsets by coupling feature learning with clustering in an iterative loop. Its core component, the YOPO (You Only Pool Once) network, is a 3D CNN specifically designed for cryo-ET data: it avoids intermediate pooling to preserve fine structural details, while a single global max-pooling layer ensures translation invariance. Rotation invariance is enforced through self-supervised training with randomly rotated subtomogram copies, and robustness to noise is achieved using Gaussian dropout. YOPO outputs 1024-dimensional feature vectors that capture both low- and high-level structural information, which are modeled as mixtures of multivariate Gaussians. Using a generalized expectation–maximization strategy with label smoothing, Bayesian information criterion model selection, and ϵ-greedy reinitialization to prevent local optima, DISCA iteratively refines both clustering assignments and feature representations until convergence. We adopt this model as the basis of our work and extend it to a semi-supervised framework.

#### Semi-supervised clustering framework.

We extend the DISCA pipeline to a semi-supervised setting for subtomogram analysis by introducing targeted enhancements that utilize limited labeled data to improve clustering stability, initialization robustness, and label reliability. Our framework retains key components from the original DISCA design, such as iterative feature extraction and network retraining, while incorporating novel clustering strategies that enable guided discovery of structural patterns. The main components of our approach are:

1. **Feature Extraction:** Following the original DISCA framework, we employ a 3D convolutional neural network (YOPO) to extract structural features from subtomograms. The network consists of ten convolutional blocks, global max pooling, and a final dense layer producing 1024-dimensional feature embeddings.
2. **Cluster Initialization via PCA Voting:** To ensure stable clustering at the outset, we introduce a multi-resolution PCA voting strategy. The semi-supervised GMM is initialized using the empirical means and covariances of labeled data for known classes. For unknown clusters, features are projected into PCA subspaces of varying dimensions (8, 16, and 32), and a semi-supervised GMM is fit in each subspace. Final assignments are determined by majority voting across projections, mitigating the instability of random initialization and integrating both supervision and unsupervised structure.
3. **Label-Guided Semi-Supervised GMM:** In subsequent iterations, we introduce a custom EM-based GMM in which labeled data are fixed to their respective classes during the E-step. To accelerate convergence and improve numerical stability, we precompute covariance matrix inverses and determinants at each iteration. Model selection is performed by minimizing the BIC, allowing dynamic discovery of the appropriate number of clusters.
4. **Confidence-Based Label Refinement:** To improve pseudo-label reliability, we estimate prediction confidence using normalized entropy over cluster responsibilities. Low-confidence samples are refined via label propagation from nearby high-confidence neighbors using cosine similarity in the feature space. This process helps correct uncertain assignments and sharpens cluster boundaries.
5. **Iterative YOPO Retraining:** As in DISCA, the YOPO network is fine-tuned after each clustering round using the updated cluster assignments. This step incorporates data augmentation and label smoothing. If the number of clusters changes, a new classification head is initialized to accommodate the revised label space. This iterative refinement ensures that learned features evolve in alignment with the clustering process.

By integrating supervision into initialization, clustering, and label refinement stages, our approach enables principled and scalable semi-supervised subtomogram classification, significantly improving the robustness and interpretability of discovered structures.

#### Label-guided GMM for semi-supervised clustering.

We introduce a semi-supervised GMM that integrates a small set of labeled subtomograms to anchor known structural classes, while allowing for the discovery of unknown clusters among unlabeled data. Let $𝐗=\{{𝐱}_{1},...,{𝐱}_{N}\}\subset {\mathbb{R}}^{d}$ denote the input feature set of *N* subtomograms, each represented in *d*-dimensional space. We consider a total of *K*_total_ clusters, of which *K*_known_ are known a priori from labeled samples. For each cluster $k\in \{1,...,K_{\text{total}}\}$, the model parameters include a mixing coefficient $\pi_{k}$, a mean vector $\mu_{k}$, and a covariance matrix $\Sigma_{k}$ that define the corresponding multivariate Gaussian distribution.

The GMM assumes that each sample ${𝐱}_{n}$ is drawn from a mixture of multivariate Gaussian distributions:


$$\begin{array}{c} p({𝐱}_{n})=\sum_{k=1}^{K_{\text{total}}}\pi_{k}\;𝒩({𝐱}_{n}|\mu_{k},\Sigma_{k}). \end{array}$$ (1)

**Partial Supervision.** Labeled samples are assigned fixed responsibilities $\gamma_{nk}=1$ for their corresponding known class *k*. Unlabeled data responsibilities are inferred through Expectation-Maximization (EM).

**E-Step.** Responsibilities $\gamma_{nk}$ are computed using:


$$\begin{array}{c} \gamma_{nk}=\frac{\pi_{k}\;𝒩({𝐱}_{n}|\mu_{k},\Sigma_{k})}{\sum_{j=1}^{K_{\text{total}}}\pi_{j}\;𝒩({𝐱}_{n}|\mu_{j},\Sigma_{j})}, \end{array}$$ (2)

with 𝒩 evaluated efficiently using precomputed $\Sigma_{k}^{-1}$ and $\det(\Sigma_{k})$ to avoid repeated inversion. Numerical stability is ensured via regularization ϵ𝐈.

**M-Step.** We update mixture weights, means, and covariances:


$$\begin{array}{c} \pi_{k}=\frac{1}{N}\sum_{n=1}^{N}\gamma_{nk},\;\mu_{k}=\frac{1}{N_{k}}\sum_{n=1}^{N}\gamma_{nk}{𝐱}_{n}, \end{array}$$ (3)

$$\begin{array}{c} \Sigma_{k}=\frac{1}{N_{k}}\sum_{n=1}^{N}\gamma_{nk}({𝐱}_{n}-\mu_{k})({𝐱}_{n}-\mu_{k}{)}^{⊤}+\epsilon 𝐈. \end{array}$$ (4)

**Cluster Initialization via PCA Voting** Unlike random initialization, unlabeled cluster means and covariances are initialized through a voting-based strategy across multiple PCA subspaces. Specifically, features are projected into subspaces of dimensions {8, 16, 32}, and clustering is performed in each subspace via semi-supervised GMM over a range of *K* values. The resulting cluster assignments are aggregated by majority voting:


$$\begin{array}{c} {\text{labels}}_{n}=\mathrm{mode}\left(\{{\hat{y}}_{n}^{\left(8\right)},{\hat{y}}_{n}^{\left(16\right)},{\hat{y}}_{n}^{\left(32\right)}\}\right). \end{array}$$ (5)

These consensus labels guide the initial assignments for unknown cluster centers, promoting consistency and reducing instability due to random starts.

**Log-Likelihood and Convergence.** We compute the complete-data log-likelihood at each iteration:


$$\begin{array}{c} \log p(𝐗|\Theta )=\sum_{n=1}^{N}\log\left(\sum_{k=1}^{K_{\text{total}}}\pi_{k}\;𝒩({𝐱}_{n}|\mu_{k},\Sigma_{k})\right), \end{array}$$ (6)

and terminate EM when the absolute change in log-likelihood is below a threshold τ.

#### Confidence-based label refinement.

To improve the quality of cluster assignments among unlabeled data, we implement a two-stage label refinement process based on entropy filtering and similarity-guided label propagation.

First, we compute the normalized entropy of each sample’s posterior responsibility vector $\gamma_{n}\in {\mathbb{R}}^{K}$ as:


$$\begin{array}{c} \text{Entropy}(\gamma_{n})=-\sum_{k=1}^{K}\gamma_{nk}\log(\gamma_{nk}+\varepsilon ), \end{array}$$ (7)

where ε is a small constant for numerical stability. The entropy values are min-max normalized:


$$\begin{array}{c} {\text{Confidence}}_{n}=1-\frac{{\text{Entropy}}_{n}-\min(\text{Entropy})}{\max(\text{Entropy})-\min(\text{Entropy})+\varepsilon }. \end{array}$$ (8)

Samples with high confidence scores (above a threshold τ=0.8) are used to propagate labels to uncertain points. We compute the cosine similarity matrix $𝐒\in {\mathbb{R}}^{N\times N}$ between feature vectors. For each low-confidence sample $x_{i}$, we identify the top-*k* most similar high-confidence neighbors (with *k* = 5), and assign a soft label by averaging their one-hot encoded labels:


$$\begin{array}{c} {\hat{𝐲}}_{i}=\frac{1}{k}\sum_{j\in {𝒩}_{k}(i)}{𝐲}_{j}, \end{array}$$ (9)

where ${𝒩}_{k}(i)$ is the set of *k* most similar confident neighbors of $x_{i}$, and ${𝐲}_{j}$ are their one-hot labels.

This process corrects uncertain assignments and enhances local cluster consistency based on feature-space similarity.

#### Evaluation metrics.

The clustering performance in this work is evaluated using four metrics: homogeneity, completeness, V-measure, and accuracy. Homogeneity, completeness, and the V-measure provide a complementary and more nuanced evaluation: homogeneity quantifies the purity of the cluster (each cluster contains only one class), completeness measures the integrity of the class (all members of a class are assigned to the same cluster), and the V-measure balances these two aspects through their harmonic mean [52]. In our application to low-SNR cryo-ET tomograms, high homogeneity ensures that structural classes are not mixed, while high completeness ensures that each class is not fragmented across multiple clusters. Together with accuracy, these metrics confirm that the semi-supervised clustering method produces both pure and complete groupings of subtomograms, even in challenging noisy and crowded environments.

Let *C* denote the set of ground-truth classes, *K* the set of predicted clusters, $a_{ck}$ the number of samples from class *c* assigned to cluster *k*, and *N* the total number of samples. We first define the entropy terms:


$$\begin{array}{c} H(C)=-\sum_{c\in C}\frac{\sum_{k\in K}a_{ck}}{N}\log\left(\frac{\sum_{k\in K}a_{ck}}{N}\right) \end{array}$$ (10)

$$\begin{array}{c} H(K)=-\sum_{k\in K}\frac{\sum_{c\in C}a_{ck}}{N}\log\left(\frac{\sum_{c\in C}a_{ck}}{N}\right) \end{array}$$ (11)

The conditional entropies are:


$$\begin{array}{ccc} H(C|K) & = & -\frac{1}{N}\sum_{k\in K}\sum_{c\in C}a_{ck}\;\log\;\left(\frac{a_{ck}}{\sum_{c^{\prime }\in C}a_{c^{\prime }k}}\right), \end{array}$$ (12)

$$\begin{array}{ccc} H(K|C) & = & -\frac{1}{N}\sum_{c\in C}\sum_{k\in K}a_{ck}\;\log\;\left(\frac{a_{ck}}{\sum_{k^{\prime }\in K}a_{ck^{\prime }}}\right). \end{array}$$ (13)

Using these definitions, the three clustering quality metrics are:


$$\begin{array}{c} \text{Homogeneity}\;h=1-\frac{H(C|K)}{H(C)} \end{array}$$ (14)

$$\begin{array}{c} \text{Completeness}\;c=1-\frac{H(K|C)}{H(K)} \end{array}$$ (15)

$$\begin{array}{c} \text{V-measure}=\frac{2\cdot h\cdot c}{h+c} \end{array}$$ (16)

The accuracy is computed after optimally mapping cluster labels to ground-truth classes (e.g., using the Hungarian algorithm):


$$\begin{array}{c} \text{Accuracy}=\frac{\sum_{i=1}^{N}1\left[y_{i}=f^{⋆}({\hat{y}}_{i})\right]}{N} \end{array}$$ (17)

where $y_{i}$ is the true label, ${\hat{y}}_{i}$ is the predicted cluster label, $f^{⋆}(\cdot )$ is the optimal mapping function, and 1[·] is the indicator function.

## References

[1] Gan L, Jensen GJ. Electron tomography of cells. Q Rev Biophys. 2012;45(1):27–56. doi: 10.1017/S0033583511000102 22082691 PMC12019784

[2] Hong Y, Song Y, Zhang Z, Li S. Cryo-Electron Tomography: The Resolution Revolution and a Surge of In Situ Virological Discoveries. Annu Rev Biophys. 2023;52:339–60. doi: 10.1146/annurev-biophys-092022-100958 36719970

[3] Turk M, Baumeister W. The promise and the challenges of cryo-electron tomography. FEBS Lett. 2020;594(20):3243–61. doi: 10.1002/1873-3468.13948 33020915

[4] Young LN, Villa E. Bringing Structure to Cell Biology with Cryo-Electron Tomography. Annu Rev Biophys. 2023;52:573–95. doi: 10.1146/annurev-biophys-111622-091327 37159298 PMC10763975

[5] Wagner J, Schaffer M, Fernández-Busnadiego R. Cryo-electron tomography-the cell biology that came in from the cold. FEBS Lett. 2017;591(17):2520–33. doi: 10.1002/1873-3468.12757 28726246

[6] Wan W. A practical look at cryo-electron tomography image processing: Key considerations for new biological discoveries. Curr Opin Struct Biol. 2025;93:103116. doi: 10.1016/j.sbi.2025.103116 40633126 PMC13200697

[7] Palovcak E, Asarnow D, Campbell MG, Yu Z, Cheng Y. Enhancing the signal-to-noise ratio and generating contrast for cryo-EM images with convolutional neural networks. IUCrJ. 2020;7(Pt 6):1142–50. doi: 10.1107/S2052252520013184 33209325 PMC7642784

[8] Pei L, Xu M, Frazier Z, Alber F. Simulating cryo electron tomograms of crowded cell cytoplasm for assessment of automated particle picking. BMC Bioinformatics. 2016;17(1):405. doi: 10.1186/s12859-016-1283-3 27716029 PMC5050594

[9] Bartesaghi A, Sprechmann P, Liu J, Randall G, Sapiro G, Subramaniam S. Classification and 3D averaging with missing wedge correction in biological electron tomography. J Struct Biol. 2008;162(3):436–50. doi: 10.1016/j.jsb.2008.02.008 18440828 PMC2556382

[10] Orlova EV, Saibil HR. Structural analysis of macromolecular assemblies by electron microscopy. Chem Rev. 2011;111(12):7710–48. doi: 10.1021/cr100353t 21919528 PMC3239172

[11] Beck M, Baumeister W. Cryo-electron tomography: can it reveal the molecular sociology of cells in atomic detail?. Trends in Cell Biology. 2016;26(11):825–37. 27671779 10.1016/j.tcb.2016.08.006

[12] Cruz-Leon S, Majtner T, Hoffmann PC, Kreysing JP, Tuijtel MW, Schaefer SL. High-confidence 3D template matching for cryo-electron tomography. bioRxiv. 2023. 10.1038/s41467-024-47839-8 PMC11088655 38734767

[13] Martinez-Sanchez A, Kochovski Z, Laugks U, Meyer Zum Alten Borgloh J, Chakraborty S, Pfeffer S, et al. Template-free detection and classification of membrane-bound complexes in cryo-electron tomograms. Nat Methods. 2020;17(2):209–16. doi: 10.1038/s41592-019-0675-5 31907446

[14] Cryo-electron tomography of E. coli minicells. Electron Microscopy Public Image Archive. EMBL-EBI. 2020. doi: 10.6019/EMPIAR-10364

[15] Cryo electron tomography of H. neapolitanus alpha-carboxysomes. Electron Microscopy Public Image Archive (EMBL-EBI). 2022. doi: 10.6019/EMPIAR-11125

[16] Watson AJI, Bartesaghi A. Advances in cryo-ET data processing: meeting the demands of visual proteomics. Curr Opin Struct Biol. 2024;87:102861. doi: 10.1016/j.sbi.2024.102861 38889501 PMC11283971

[17] Liu S, Ma Y, Ban X, Zeng X, Nallapareddy V, Chaudhari A. Efficient cryo-electron tomogram simulation of macromolecular crowding with application to sars-cov-2. In: 2020 IEEE International Conference on Bioinformatics and Biomedicine (BIBM), 2020. 80–7.

[18] Hecksel CW, Darrow MC, Dai W, Galaz-Montoya JG, Chin JA, Mitchell PG, et al. Quantifying Variability of Manual Annotation in Cryo-Electron Tomograms. Microsc Microanal. 2016;22(3):487–96. doi: 10.1017/S1431927616000799 27225525 PMC5111626

[19] Du X, Wang H, Zhu Z, Zeng X, Chang Y-W, Zhang J, et al. Active learning to classify macromolecular structures in situ for less supervision in cryo-electron tomography. Bioinformatics. 2021;37(16):2340–6. doi: 10.1093/bioinformatics/btab123 33620460 PMC12158443

[20] Liu S, Du X, Xi R, Xu F, Zeng X, Zhou B. Semi-supervised macromolecule structural classification in cellular electron cryo-tomograms using 3D autoencoding classifier. In: BMVC, 2019.

[21] Huang Q, Zhou Y, Liu HF, Bartesaghi A. Accurate detection of proteins in cryo-electron tomograms from sparse labels. In: European Conference on Computer Vision. Springer. 2022. 644–60.

[22] Liu Z, Yuan C, Zhang Z, Zhou X, Li X, Tian Z, et al. A hybrid YOLO-UNet3D framework for automated protein particle annotation in Cryo-ET images. Sci Rep. 2025;15(1):25033. doi: 10.1038/s41598-025-09522-w 40646021 PMC12254289

[23] Purnell C, Heebner J, Nguyen L, Swulius MT, Hylton R, Kabonick S, et al. Training Generalized Segmentation Networks with Real and Synthetic Cryo-ET data. bioRxiv. 2025.

[24] Chen M, Dai W, Sun SY, Jonasch D, He CY, Schmid MF, et al. Convolutional neural networks for automated annotation of cellular cryo-electron tomograms. Nat Methods. 2017;14(10):983–5. doi: 10.1038/nmeth.4405 28846087 PMC5623144

[25] Zeng X, Kahng A, Xue L, Mahamid J, Chang Y-W, Xu M. High-throughput cryo-ET structural pattern mining by unsupervised deep iterative subtomogram clustering. Proc Natl Acad Sci U S A. 2023;120(15):e2213149120. doi: 10.1073/pnas.2213149120 37027429 PMC10104553

[26] Powell BM, Davis JH. Learning structural heterogeneity from cryo-electron sub-tomograms with tomoDRGN. Nat Methods. 2024;21(8):1525–36. doi: 10.1038/s41592-024-02210-z 38459385 PMC11655136

[27] Moebel E, Kervrann C. Towards unsupervised classification of macromolecular complexes in cryo electron tomography: Challenges and opportunities. Comput Methods Programs Biomed. 2022;225:107017. doi: 10.1016/j.cmpb.2022.107017 35901628

[28] Huang Q, Zhou Y, Bartesaghi A. MiLoPYP: self-supervised molecular pattern mining and particle localization in situ. Nat Methods. 2024;21(10):1863–72. doi: 10.1038/s41592-024-02403-6 39251798 PMC11468773

[29] Lin R, Zeng X, Kitani K, Xu M. Adversarial domain adaptation for cross data source macromolecule in situ structural classification in cellular electron cryo-tomograms. Bioinformatics. 2019;35(14):i260–8. doi: 10.1093/bioinformatics/btz364 31510673 PMC6612867

[30] Chen Y, Pfeffer S, Fernández JJ, Sorzano COS, Förster F. Autofocused 3D classification of cryoelectron subtomograms. Structure. 2014;22(10):1528–37. doi: 10.1016/j.str.2014.08.007 25242455

[31] Galaz-Montoya JG. The advent of preventive high-resolution structural histopathology by artificial-intelligence-powered cryogenic electron tomography. Front Mol Biosci. 2024;11:1390858. doi: 10.3389/fmolb.2024.1390858 38868297 PMC11167099

[32] Xu M, Singla J, Tocheva EI, Chang Y-W, Stevens RC, Jensen GJ, et al. De Novo Structural Pattern Mining in Cellular Electron Cryotomograms. Structure. 2019;27(4):679-691.e14. doi: 10.1016/j.str.2019.01.005 30744995 PMC7542605

[33] Zeng X, Xu M. AITom: Open-source AI platform for cryo-electron tomography data analysis. arXiv preprint. 2019. doi: 10.48550/arXiv.191103044

[34] Heebner JE, Purnell C, Hylton RK, Marsh M, Grillo MA, Swulius MT. Deep Learning-Based Segmentation of Cryo-Electron Tomograms. J Vis Exp. 2022;(189):10.3791/64435. doi: 10.3791/64435 36440884

[35] Zidovska J. CryoET Object Identification Challenge Dataset. 2023. https://cryoetdataportal.czscience.com/datasets/10446

[36] Martinez-Sanchez A, Lamm L, Jasnin M, Phelippeau H. Simulating the Cellular Context in Synthetic Datasets for Cryo-Electron Tomography. IEEE Trans Med Imaging. 2024;43(11):3742–54. doi: 10.1109/TMI.2024.3398401 38717878

[37] Ermel U, Cheng A, Ni JX, Gadling J, Venkatakrishnan M, Evans K, et al. A data portal for providing standardized annotations for cryo-electron tomography. Nat Methods. 2024;21(12):2200–2. doi: 10.1038/s41592-024-02477-2 39433879

[38] Iudin A, Korir PK, Somasundharam S, Weyand S, Cattavitello C, Fonseca N, et al. EMPIAR: the Electron Microscopy Public Image Archive. Nucleic Acids Res. 2023;51(D1):D1503–11. doi: 10.1093/nar/gkac1062 36440762 PMC9825465

[39] Dhakal A, Gyawali R, Wang L, Cheng J. CryoPPP: a large expert-labelled cryo-EM image dataset for machine learning protein particle picking. bioRxiv. 2023. 10.1038/s41597-023-02280-2 PMC10287764 37349345

[40] Chebli A, Djebbar A, Marouani HF. Semi-Supervised Learning for Medical Application: A Survey. In: 2018 International Conference on Applied Smart Systems (ICASS), 2018. 1–9. 10.1109/icass.2018.8651980

[41] Gupta T, He X, Uddin MR, Zeng X, Zhou A, Zhang J, et al. Self-supervised learning for macromolecular structure classification based on cryo-electron tomograms. Front Physiol. 2022;13:957484. doi: 10.3389/fphys.2022.957484 36111160 PMC9468634

[42] Kremer JR, Mastronarde DN, McIntosh JR. Computer visualization of three-dimensional image data using IMOD. J Struct Biol. 1996;116(1):71–6. doi: 10.1006/jsbi.1996.0013 8742726

[43] Tang G, Peng L, Baldwin PR, Mann DS, Jiang W, Rees I, et al. EMAN2: an extensible image processing suite for electron microscopy. J Struct Biol. 2007;157(1):38–46. doi: 10.1016/j.jsb.2006.05.009 16859925

[44] Pettersen EF, Goddard TD, Huang CC, Meng EC, Couch GS, Croll TI, et al. UCSF ChimeraX: Structure visualization for researchers, educators, and developers. Protein Sci. 2021;30(1):70–82. doi: 10.1002/pro.3943 32881101 PMC7737788

[45] Last MGF, Abendstein L, Voortman LM, Sharp TH. Streamlining segmentation of cryo-electron tomography datasets with Ais. Elife. 2024;13:RP98552. doi: 10.7554/eLife.98552 39704648 PMC11661790

[46] Lamm L, Zufferey S, Righetto RD, Wietrzynski W, Yamauchi KA, Burt A. Membrain v2: an end-to-end tool for the analysis of membranes in cryo-electron tomography. biorxiv. 2024.

[47] Berg S, Kutra D, Kroeger T, Straehle CN, Kausler BX, Haubold C, et al. ilastik: interactive machine learning for (bio)image analysis. Nat Methods. 2019;16(12):1226–32. doi: 10.1038/s41592-019-0582-9 31570887

[48] Pennington A, King ONF, Tun WM, Ho EML, Luengo I, Darrow MC, et al. SuRVoS 2: Accelerating Annotation and Segmentation for Large Volumetric Bioimage Workflows Across Modalities and Scales. Front Cell Dev Biol. 2022;10:842342. doi: 10.3389/fcell.2022.842342 35433703 PMC9011330

[49] Castaño-Díez D, Kudryashev M, Arheit M, Stahlberg H. Dynamo: a flexible, user-friendly development tool for subtomogram averaging of cryo-EM data in high-performance computing environments. J Struct Biol. 2012;178(2):139–51. doi: 10.1016/j.jsb.2011.12.017 22245546

[50] RCSB Protein Data Bank Website. 2025. https://www.rcsb.org/

[51] Wriggers W. Conventions and workflows for using Situs. Biological crystallography. 2012;68(4):344–51. 22505255 10.1107/S0907444911049791 PMC3322594

[52] Bonnin R. Machine Learning for Developers: Uplift Your Regular Applications with the Power of Statistics, Analytics, and Machine Learning. Packt Publishing Ltd. 2017.

