# Supplementary material for: Semi-supervised clustering with knowledge-guided representation learning in cryo-electron tomography
Source: PLOS Digit Health. 2026 Aug 31;5(8):e0001619. doi: 10.1371/journal.pdig.0001619 (PMC13529008; doi:10.1371/journal.pdig.0001619)
Supplement: Supplementary file 1 [file pdig.0001619.s002.pdf]

Response to Reviewers

We are very grateful to the Academic Editor, the Editor-in-Chief, and both reviewers for their careful evaluation of our manuscript and for their constructive and insightful comments. We sincerely appreciate the time and effort invested in reviewing our work. We have revised the manuscript accordingly and have made substantial additions to address the concerns raised, including broader semi-supervised comparisons, additional ablation studies, sensitivity analyses, real-data unknown-class discovery experiments, clearer methodological explanations, and improved reproducibility and data-availability details.

Please find our point-by-point response below. In the following response, "Q:" indicates the original comments from the editor or reviewers, shown in blue, and "A:" indicates our responses, shown in black.

Reviewer #1

Q: The discovery capability was demonstrated by leaving five classes unlabled in the simulated data. However, I do not see such demonstration in the real dataset. Could this be done?

A: We thank the reviewer for this important suggestion. We agree that demonstrating unknown-structure discovery on experimental data is important, because the central goal of our method is not only to improve clustering with partial supervision, but also to preserve the ability to identify structures that are absent from the labeled subset.

To address this point, we added a new experiment using the experimental Dragonfly cryo-ET dataset. We designed this experiment as a real-data analogue of the simulated unknown-class setting. Specifically, we used a leave-one-class-unlabeled protocol: in each run, one structural class was completely withheld from the labeled subset, while the remaining classes contributed only 1% labeled examples. The withheld class therefore had no labeled examples during training or clustering, and successful recovery of this class reflects the model's ability to discover an unknown structure from the unlabeled data. We repeated this procedure for all six structural classes so that each class served once as the fully unlabeled unknown class.

We added the new experiment to the revised manuscript under the section "Performance on Dragonfly Cryo-ET dataset." We also added a new table reporting the recovery accuracy for each fully unlabeled class:

Response Table R1. Structural discovery on the experimental Dragonfly cryo-ET dataset.

Fully unlabeled class	Number of subtomograms	Recovery accuracy
Membrane	1,294	0.65
Actin	4,641	0.23
Cofilactin	1,042	0.31
Microtubule	318	0.61
Ribosome	484	0.74
TriC	253	0.52

These results demonstrate that the proposed framework can recover several fully unseen structures in experimental cryo-ET data. In particular, Ribosome, Membrane, and Microtubule were recovered with accuracies of 0.74, 0.65, and 0.61, respectively, despite being completely absent from the labeled subset in their corresponding runs. TriC was recovered with moderate accuracy of 0.52. These findings support the discovery capability of the model in a real-data setting: the method does not simply classify unlabeled samples into known categories, but can form clusters corresponding to structures that were not represented among the labeled examples.

We also discuss the more difficult cases in the revised manuscript. Actin and Cofilactin showed lower recovery accuracies of 0.23 and 0.31, respectively. We believe this is likely due to their strong structural similarity, since both are filament-associated classes and may occupy nearby regions in the learned feature space. When one of these classes is withheld from supervision, the model may assign some of its subtomograms to the other filament-like class or split them across related clusters. This result provides a realistic limitation of unknown-structure discovery in experimental cryo-ET: discovery is more reliable when the unknown class is structurally distinctive, and more challenging when the unseen class is morphologically similar to a labeled class.

Q: The proposed method was compared against the original DISCA model but would it be possible to also compare to other semi-supervised methods? These two simulations would strenghten the manuscript. Or I suggest that the authors include rationale on why they restrict to these comparisons.

A: We thank the reviewer for raising this important point. We agree that comparison only against the original unsupervised DISCA model is not sufficient to determine whether the observed gains come from our proposed extension specifically, or simply from the general benefit of adding a small labeled subset. We have therefore expanded the experimental evaluation in the revised manuscript.

Before describing the new experiments, we would like to clarify the comparator choice. To our knowledge, there is currently no cryo-ET method that directly combines semi-supervised clustering with unknown structural discovery. Existing semi-supervised or weakly supervised cryo-ET methods are primarily classification, detection, or segmentation frameworks that assume predefined target classes, whereas discovery-oriented approaches such as DISCA are mainly unsupervised. Therefore, a directly equivalent baseline is not currently available. To address this limitation as fairly as possible, we compared our method against the original DISCA model, a DISCA variant with the true number of clusters fixed, generic semi-supervised clustering baselines, and a sequence of internal ablations of our own method.

Specifically, we added two generic semi-supervised clustering baselines that use the same labeled subset as our method: seeded k-means and COP-k-means. Seeded k-means tests whether label-informed centroid initialization alone is sufficient to explain the improvement, while COP-k-means tests whether standard pairwise supervision through must-link and cannot-link constraints is sufficient. We also added a sequence of ablations to isolate the contribution of each proposed component: label-anchored GMM (LA-GMM), LA-GMM with PCA-voting initialization, LA-GMM with PCA-voting initialization and confidence filtering, and the full model with label propagation. This design separates the general effect of sparse supervision from the specific contribution of label anchoring, PCA voting, confidence scoring, and label propagation.

The expanded comparison is reported in Table 1 of our revised manuscript, reproduced below for clarity.

The results show that the generic semi-supervised baselines did not reproduce the gains of the proposed method. Seeded k-means performed poorly under difficult imaging conditions, reaching only 0.07 accuracy at SNR = 0.01 and $\pm 40^\circ$ tilt. COP-k-means improved over seeded k-means in some settings, but still remained below the proposed GMM-based variants. For example, in the same SNR = 0.01, $\pm 40^\circ$ setting, COP-k-means achieved 0.13 accuracy, whereas LA-GMM reached 0.47 accuracy and LA-GMM+PCA reached 0.48 accuracy. These results indicate that sparse labels alone, when incorporated through standard semi-supervised clustering mechanisms, are not sufficient to explain the observed improvements.

The ablation sequence further shows that the gains come from the proposed integration of sparse labels into the DISCA framework. LA-GMM substantially improved over seeded and constrained k-means, indicating that probabilistic label anchoring is more effective than centroid seeding or pairwise constraints in this setting. Adding PCA voting further improved performance, especially at SNR = 0.03, where accuracy increased from 0.79 to 0.87 under $\pm 60^\circ$ tilt and from 0.72 to 0.85 under $\pm 40^\circ$ tilt. Adding confidence filtering further improved the SNR = 0.03 condition, reaching 0.93 accuracy under $\pm 60^\circ$ tilt and 0.87 under $\pm 40^\circ$ tilt. The full model achieved the best or tied-best performance in most conditions, including 0.98 accuracy at SNR = 0.10 and $\pm 60^\circ$ tilt, 0.96 at SNR = 0.03 and $\pm 60^\circ$ tilt, and 0.94 at SNR = 0.10 and $\pm 40^\circ$ tilt.

Importantly, the new experiments also revealed a limitation under the most extreme imaging condition. At SNR = 0.01 with $\pm 40^\circ$ tilt, the simpler LA-GMM and LA-GMM+PCA variants outperformed the full model. This suggests that entropy-based confidence scoring and label propagation can become less reliable when the feature space is severely degraded by noise and missing-wedge artifacts. In such cases, a low-entropy GMM assignment may still be incorrect, and label propagation can amplify incorrect pseudo-labels by spreading them to neighboring samples. We therefore now interpret confidence scoring and label propagation as useful refinement mechanisms in most settings, but not universally beneficial under extreme low-SNR, limited-tilt conditions.

Response Table R2. Performance results on synthetic datasets simulated via AITom tools (True $k = 23$).

**SNR**	**Simulated ±60°**			**Simulated ±40°**		
	0.10	0.030	0.010	0.10	0.030	0.010
**DISCA**						
k	20	20	20	23	20	20
Homogeneity ↑	0.78	0.54	0.48	0.92	0.81	0.24
Completeness ↑	0.85	0.59	0.57	0.94	0.87	0.25
V-measure ↑	0.82	0.56	0.52	0.93	0.84	0.25
Accuracy ↑	–	–	–	0.85	–	–
**DISCA (fixed $k = 23$)**						
k	23	23	23	23	23	23
Homogeneity ↑	0.91	0.74	0.50	0.92	0.83	0.37
Completeness ↑	0.91	0.78	0.51	0.94	0.87	0.39
V-measure ↑	0.91	0.76	0.51	0.93	0.85	0.38
Accuracy ↑	0.92	0.57	0.35	0.85	0.72	0.25
**Seeded k-means (fixed $k = 23$)**						
k	23	23	23	23	23	23
Homogeneity ↑	0.65	0.54	0.12	0.58	0.21	0.09
Completeness ↑	0.66	0.59	0.13	0.59	0.19	0.09
V-measure ↑	0.65	0.56	0.12	0.59	0.20	0.09
Accuracy ↑	0.55	0.49	0.11	0.45	0.19	0.07
**COP-k-means (fixed $k = 23$)**						
k	23	23	23	23	23	23
Homogeneity ↑	0.70	0.54	0.21	0.77	0.37	0.16
Completeness ↑	0.73	0.61	0.22	0.78	0.30	0.17
V-measure ↑	0.72	0.57	0.21	0.78	0.33	0.17
Accuracy ↑	0.58	0.32	0.17	0.72	0.39	0.13
**Ours w/ LA-GMM**						
k	23	23	23	23	23	23
Homogeneity ↑	0.96	0.80	0.56	0.91	0.72	0.62
Completeness ↑	**0.99**	0.82	0.57	0.92	0.74	0.67
V-measure ↑	**0.98**	0.81	0.57	0.92	0.73	**0.65**
Accuracy ↑	0.90	0.79	0.54	0.88	0.72	0.47
**Ours w/ LA-GMM + PCA**						
k	23	23	23	23	23	23
Homogeneity ↑	0.97	0.90	0.58	0.91	0.83	**0.63**
Completeness ↑	**0.99**	0.92	0.58	0.92	0.84	**0.68**
V-measure ↑	**0.98**	0.91	0.58	0.92	0.84	**0.65**
Accuracy ↑	0.92	0.87	**0.56**	0.90	0.85	**0.48**
**Ours w/ LA-GMM + PCA + Confidence**						
k	23	23	23	23	23	23
Homogeneity ↑	0.97	0.93	0.58	0.92	0.85	0.62
Completeness ↑	**0.98**	0.93	0.59	0.93	**0.86**	0.67
V-measure ↑	**0.98**	0.93	0.59	0.93	**0.86**	0.64
Accuracy ↑	0.95	0.93	0.54	0.90	0.87	**0.48**
**Our Full Model**						
k	23	23	23	23	23	23
Homogeneity ↑	**0.98**	**0.94**	**0.61**	**0.94**	**0.86**	0.52
Completeness ↑	**0.98**	**0.94**	**0.62**	**0.94**	**0.86**	0.56
V-measure ↑	**0.98**	**0.94**	**0.61**	**0.94**	**0.86**	0.54
Accuracy ↑	**0.98**	**0.96**	**0.56**	**0.94**	**0.88**	0.41

Q: Sensitivity analyses are lacking overall. Specifically, why 1% is chosen to enable semi-supervised learning? why not other proportions? Please explain the rationale or conduct sensitivity analyses for the parameters used in the models.

A: We thank the reviewer for this important suggestion. We agree that relying only on a single labeled-data proportion does not fully characterize the robustness of the method or justify the choice of 1% supervision. To address this concern, we added a new sensitivity analysis section to the revised manuscript. The sensitivity analysis evaluates three key choices in the model: (i) the proportion of labeled subtomograms, (ii) the number of high-confidence neighbors used during confidence-based label propagation, and (iii) the entropy-confidence threshold used to define high-confidence samples.

We performed these analyses under an intentionally difficult condition using a 23-class simulated subtomogram dataset generated at SNR = 0.001 with a $\pm 40^\circ$ missing-wedge tilt range. This setting was selected to approximate the severe noise and limited angular sampling encountered in experimental cryo-ET data. The goal was to test whether the method remains robust under a low-supervision and low-quality imaging regime, rather than only under favorable simulated conditions.

The sensitivity results are summarized in Response Figure R1.

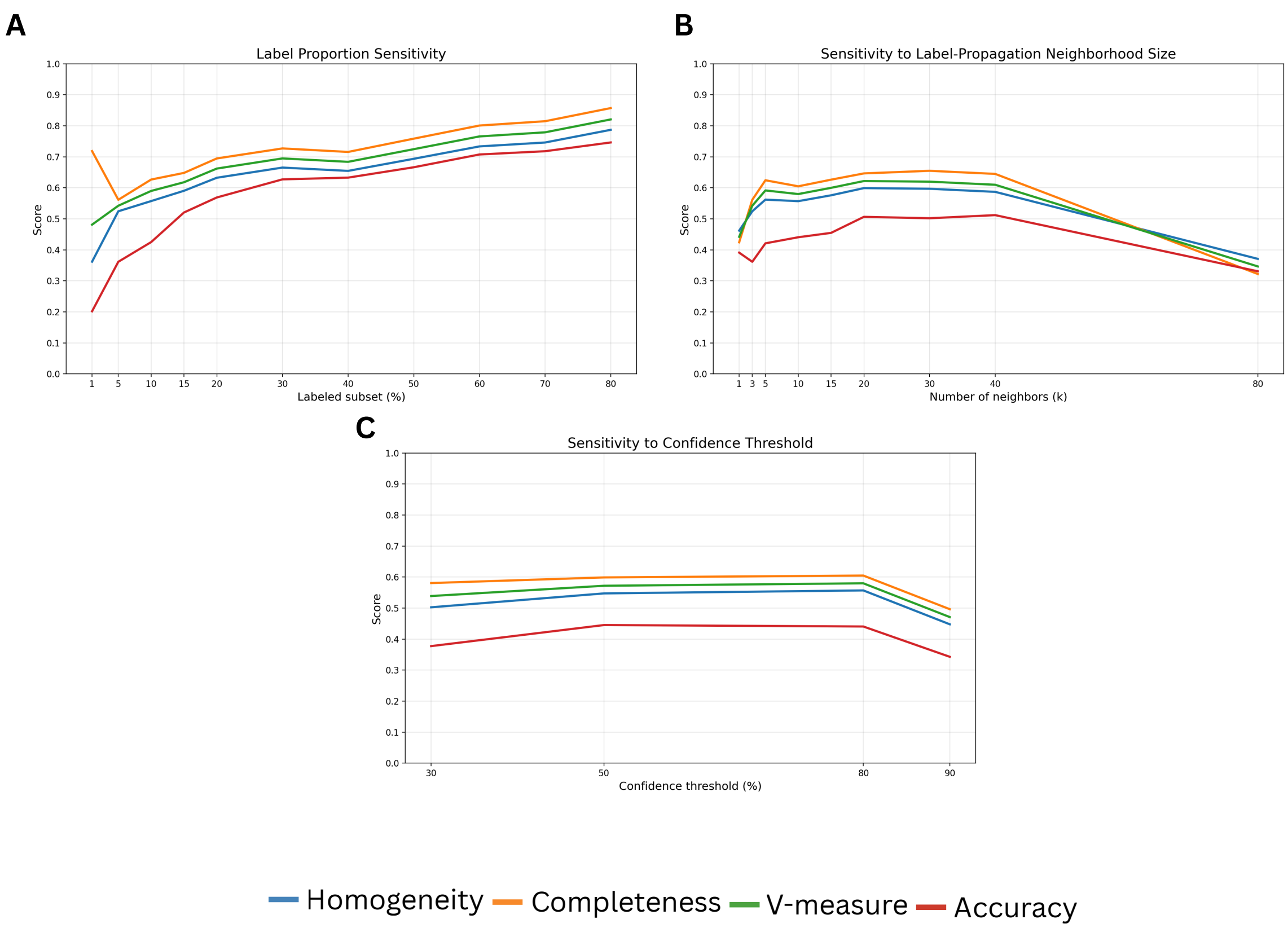

Response Figure R1. Sensitivity analysis under extreme low-SNR simulated conditions. Sensitivity analyses were performed on a 23-class simulated subtomogram dataset generated at SNR = 0.001 with a $\pm 40^\circ$ missing-wedge tilt range. (A) Effect of increasing the labeled subset from 1% to 80%. Performance improves with additional labels, supporting the use of 1% as a conservative low-supervision setting rather than an optimal label fraction. (B) Effect of the number of high-confidence neighbors used in confidence-based label propagation. Moderate neighborhood sizes improve performance, whereas very large neighborhoods lead to oversmoothing. (C) Effect of the entropy-confidence threshold used to define high-confidence samples. A threshold of 0.80 provides the best V-measure, while 0.90 is overly restrictive. Blue, orange, green, and red curves denote homogeneity, completeness, V-measure, and accuracy, respectively.

For the labeled subset proportion analysis, we varied the labeled fraction from 1% to 80%. As expected, performance improved as more labeled examples were provided. Accuracy increased from 0.20 at 1% labeled data to 0.75 at 80%, while V-measure increased from 0.48 to 0.82. Homogeneity also increased from 0.36 to 0.79. These results confirm that additional supervision improves clustering quality by providing stronger anchors for the known classes. However, the purpose of using 1% in the main experiments was not to claim that 1% is optimal. Rather, 1% was chosen as a deliberately stringent low-annotation setting, motivated by the practical reality that expert annotation in cryo-ET is expensive and time-consuming. Therefore, the 1% setting serves as a conservative test of whether the proposed framework can operate when only minimal expert labels are available.

We also evaluated the number of high-confidence neighbors used during confidence-based label propagation, testing $k = \{1, 3, 5, 10, 15, 20, 30, 40, 80\}$. Using only one neighbor gave weak performance, with V-measure of 0.44 and accuracy of 0.39, suggesting that single-neighbor propagation is sensitive to local noise. Performance improved with moderate neighborhood sizes, reaching the best V-measure at $k = 20$ and the best accuracy at $k = 40$. However, performance dropped sharply at $k = 80$, where V-measure decreased to 0.35 and accuracy to 0.33. This indicates that very large neighborhoods oversmooth labels across nearby clusters, while moderate neighborhoods provide a better balance between local robustness and class specificity. Our main experiments used $k = 5$ as a conservative local-neighborhood setting, and the sensitivity analysis shows that the method remains stable over a range of moderate neighborhood sizes.

Finally, we evaluated the entropy-confidence threshold used to define high-confidence samples, testing thresholds of 0.30, 0.50, 0.80, and 0.90. Performance improved from threshold 0.30 to 0.80, with V-measure increasing from 0.54 to 0.58. However, increasing the threshold to 0.90 reduced performance, with V-measure decreasing to 0.47 and accuracy to 0.34. This suggests that too low a threshold may allow unreliable pseudo-labels to propagate, whereas too high a threshold can leave too few confident samples for effective refinement. A threshold of 0.80 therefore provides a conservative but not overly restrictive setting and was used as the default value in the main experiments.

Q: On the minor side, for Figure 2, have you verified the separation in t-SNE with UMAP or PCA plots to ensure that this is not an artifact?

A: We thank the reviewer for this helpful suggestion. We agree that t-SNE visualizations can sometimes be overinterpreted and that the apparent feature separation should be checked using an additional embedding method.

To address this concern, we performed an additional verification experiment. We simulated a new 23-class subtomogram dataset under the same representative condition used for Figure 2 in the manuscript, namely SNR $= 0.03$ with a $\pm 60^\circ$ missing-wedge tilt range. We then compared the learned feature representations from our semi-supervised method and the unsupervised DISCA baseline using both t-SNE and UMAP across iterations 1, 2, and 5. Panel A corresponds to our semi-supervised method, while Panel B corresponds to the unsupervised DISCA baseline.

The additional visualization is shown below. The UMAP plots support the same qualitative conclusion as the t-SNE plots reported in the manuscript. In Panel A, our method forms more compact and separated feature groups from the early iterations, and the clusters remain more clearly organized through later iterations. In contrast, Panel B shows stronger class mixing for the unsupervised DISCA baseline, especially in the early iterations, with less stable separation across the embedding space. This agreement between t-SNE and UMAP indicates that the improved separation observed in Figure 2 is not simply a t-SNE artifact, but reflects a more organized feature representation learned by the proposed label-aware framework.

Due to space limitations, we did not add this additional UMAP/t-SNE comparison figure to the revised manuscript. However, we include it in this response to directly address the reviewer's concern and to show that the representation-level conclusion is consistent across embedding methods.

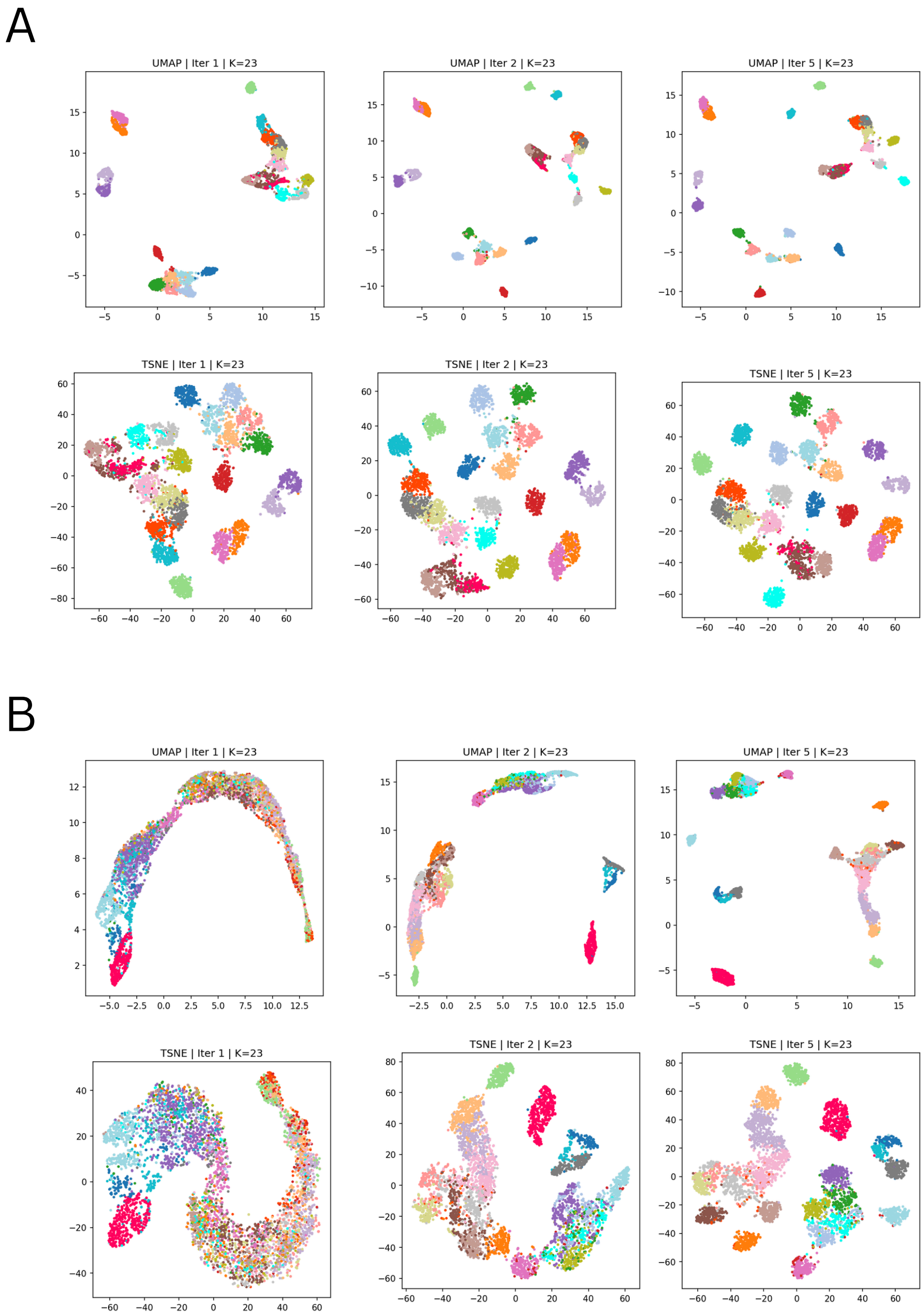

Response Figure R2. Additional verification of feature-space separation using UMAP and t-SNE. A new 23-class simulated subtomogram dataset was generated at SNR = 0.03 with a ±60° missing-wedge tilt range. Panel A shows the embeddings obtained using our semi-supervised method, while Panel B shows the embeddings obtained using the unsupervised DISCA baseline. For each method, UMAP and t-SNE visualizations are shown at iterations 1, 2, and 5. The UMAP plots show the same qualitative trend as t-SNE: our method produces more compact and separated feature groups from early iterations, whereas the unsupervised DISCA baseline shows stronger class mixing and less stable separation.

Reviewer #2

Q: Overall, the direction is compelling and potentially useful for structural discovery in cryo-ET where partial labels are common. However, several methodological clarifications and additional robustness evaluations are needed to support the strongest claims and to ensure reproducibility.

A: We thank the reviewer for this constructive assessment. We agree that the original manuscript needed clearer methodological explanation, additional robustness evaluation, and stronger reproducibility details. In response, we made several substantial revisions throughout the manuscript. The main revisions are summarized below, with references to the corresponding tables and figures included in this response.

First, we expanded the comparative evaluation to address robustness and attribution of the gains. As shown in Response Table 2 in this response, the revised manuscript now includes a broader comparison beyond unsupervised DISCA. In addition to the original DISCA baseline and the previously included DISCA variant with the true number of clusters fixed, we added seeded k-means and COP-k-means, both evaluated under the same conditions. We also added a stepwise ablation sequence: LA-GMM, LA-GMM+PCA, LA-GMM+PCA+confidence, and the full model with label propagation. These additions directly test whether the observed improvement comes simply from adding labels or from the proposed integration of sparse labels into the DISCA framework. The results show that seeded k-means and COP-k-means do not reproduce the gains of our method, while the ablations show that label anchoring, PCA voting, confidence filtering, and label propagation each affect performance. Importantly, the ablations also reveal a realistic limitation: under the most extreme SNR = 0.01, $\pm 40^\circ$ condition, the simpler LA-GMM and LA-GMM+PCA variants outperform the full model, suggesting that entropy-based confidence and label propagation can become unreliable when the feature space is severely degraded by noise and missing-wedge artifacts.

Second, we added a real-data unknown-structure discovery experiment using the Dragonfly cryo-ET dataset. As shown in Response Table 1 in this response, we introduced a leave-one-class-unlabeled protocol on experimental Dragonfly subtomograms. In each run, one structural class was completely withheld from the labeled subset, while the remaining classes contributed only 1% labeled examples. This was repeated for each class. The results show that the method can recover several fully unseen structures in real cryo-ET data, including Ribosome, Membrane, and Microtubule, while also revealing realistic limitations for structurally similar classes such as Actin and Cofilactin. This directly strengthens the discovery claim beyond simulation.

Third, we added a new sensitivity analysis section to evaluate key robustness questions. As shown in Response Figure R1 in this response, we varied the labeled subset proportion, the number of neighbors used for confidence-based label propagation, and the entropy-confidence threshold. These analyses were performed under an intentionally difficult setting, SNR = 0.001 with a $\pm 40^\circ$ missing-wedge tilt range. The results show that performance improves with more labeled data, as expected, but also clarify that the 1% setting was chosen as a deliberately conservative low-annotation regime rather than an optimal label fraction. The sensitivity analysis also shows that moderate neighborhood sizes and a confidence threshold of 0.80 work best, while overly broad propagation or overly strict filtering can reduce

performance under extreme low-SNR conditions.

Fourth, we addressed the representation-level robustness concern raised by Reviewer #1, which is also relevant to Reviewer #2's request for stronger support of the claims. As shown in Response Figure R2 in this response, we simulated a new 23-class dataset at SNR = 0.03 with a $\pm 60^{\circ}$ missing-wedge tilt range and compared both UMAP and t-SNE embeddings for our method and the unsupervised DISCA baseline. The UMAP plots show the same qualitative trend as t-SNE: our semi-supervised method produces more compact and separated feature groups from early iterations, while unsupervised DISCA shows stronger mixing and less stable separation. This supports the conclusion that the observed representation-level improvement is not only a t-SNE artifact.

Finally, we added a dedicated Reproducibility and Data Availability section. This section points to the public GitHub repository containing the source code, example commands, environment requirements, input-file descriptions, and expected outputs. It also explains that the processed datasets are provided through Hugging Face links listed in the repository, including the AITom-generated synthetic subtomogram dataset, the processed Dragonfly cryo-ET dataset, and the PolNet-generated synthetic tomograms, while the CZII data are available through the CryoET Data Portal: https://github.com/Kassab11/Semi-Supervised-Clustering-with-Knowledge-Guided-Representation-Learning-in-Cryo-Electron-Tomography.
