# Supplementary material for: Semi-supervised clustering with knowledge-guided representation learning in cryo-electron tomography
Source: PLOS Digit Health. 2026 Aug 31;5(8):e0001619. doi: 10.1371/journal.pdig.0001619 (PMC13529008; doi:10.1371/journal.pdig.0001619)
Supplement: Peer review history

# Peer review: Decision Letter 0

**DOI:** 10.1371/journal.pdig.0001619.r001

**Authors:** Ismini Lourentzou, Satyananda Kashyap

17 Mar 2026

Response to Reviewers Revised Manuscript with Track Changes Manuscript **Journal Requirements:**

1. When completing the data availability statement of the submission form, you indicated that you will make your data available on acceptance. We strongly recommend all authors decide on a data sharing plan before acceptance, as the process can be lengthy and hold up publication timelines. Please note that, though access restrictions are acceptable now, your entire data will need to be made freely accessible if your manuscript is accepted for publication. This policy applies to all data except where public deposition would breach compliance with the protocol approved by your research ethics board. If you are unable to adhere to our open data policy, please kindly revise your statement to explain your reasoning and we will seek the editor's input on an exemption. Please be assured that, once you have provided your new statement, the assessment of your exemption will not hold up the peer review process.

2. We ask that a manuscript source file is provided at Revision. Please upload your manuscript file as a .doc, .docx, .rtf or .tex.

3. Please upload a copy of Figure 1-6 which you refer to in your text on pages 3,6,7,8,11. Or, if the figure is no longer to be included as part of the submission please remove all reference to it within the text.

**Additional Editor Comments (if provided):**

Both reviewers view the work positively in principle, and I agree that there is a publishable paper here. I also agree that the current manuscript would benefit from a more substantial revision before it is ready for publication. Please look at the reviewers' comments for detailed information, but here is the summary of the major points that are being asked to be revised.

1. The main unresolved issue is attribution of the empirical gains.

The central question is not whether partial supervision can help; that seems plausible, and the manuscript provides encouraging evidence in that direction. The more specific question is whether the current experiments establish that the proposed extension to DISCA itself is responsible for the observed gains. In its current form, the paper mainly shows that the full proposed method outperforms unsupervised DISCA. This leaves open the possibility that a substantial part of the improvement could arise from the general benefit of incorporating a small amount of labeled data, rather than from the specific combination of label anchoring, PCA-voting initialization, confidence scoring, and label propagation introduced here. Reviewer 1’s request for broader comparison is therefore well taken. I agree that this is a central issue. The manuscript would be strengthened either by broader comparison to other semi-supervised alternatives or by a clearer rationale for why DISCA is the only meaningful comparator in this setting.

2. Stronger analysis around the low-label regime: the reviewers also mentioned why 1% was chosen to enable semi-supervised learning and why not take other proportions. Since the practical motivation of this paper rests on the performance in low-label settings. Reliance on a single label fraction leaves an important part of the story underexplored. A sensitivity analysis across the different label fractions would strengthen the paper's claim.

3. The discovery claim is compelling in simulation, but less well demonstrated on real data.

An attractive feature of the manuscript is its attempt to preserve discovery of unseen classes rather than reducing the problem to ordinary semi-supervised classification. R1 correctly notes that this is demonstrated in the simulated setting by leaving several cases unlabeled, but it's not shown as clearly on the real datasets. I think I agree that the manuscript would be stronger if either provided a closer real-data analogue of this discovery setting or more carefully reconsider's the scope of this claim.

4. The representation-level evidence should be supported more cautiously.

The suggestion to verify the t-SNE separation with UMAP or PCA is reasonable. Because embedding visualizations can be overinterpreted, it would be helpful to show that the apparent structure is not specific to one visualization method.

More generally, consistent with Reviewer 2’s comments, the manuscript would also benefit from clearer methodological explanation and stronger reproducibility detail. Some design choices and robustness considerations would profit from fuller discussion so that readers can better assess the reliability and generality of the proposed approach.

In my view, the paper would be materially strengthened by:

• broader comparative evaluation, or a stronger rationale for comparator choice

• sensitivity analyses around the labeled-data regime and key design choices

• stronger support for the discovery claim beyond the simulated setting

• clearer methodological and reproducibility detail

**Reviewers' Comments:**

**Comments to the Author**

1. Does this manuscript meet PLOS Digital Health’s publication criteria?

Reviewer #1: Yes

Reviewer #2: Partly

**********

2. Has the statistical analysis been performed appropriately and rigorously?

Reviewer #1: Yes

Reviewer #2: Yes

**********

3. Have the authors made all data underlying the findings in their manuscript fully available (please refer to the Data Availability Statement at the start of the manuscript PDF file)?

The PLOS Data policy

Reviewer #1: Yes

Reviewer #2: No

**********

4. Is the manuscript presented in an intelligible fashion and written in standard English?

Reviewer #1: Yes

Reviewer #2: Yes

**********

Reviewer #1: The problem is well-motivated. Cryo-ET labeling would benefit greatly from semi-supervised learning. Incorporating biological knowledge is a clearly a logical and promising step. I congratulate the authors on producing this rigorous work.

Three questions on the methods arose. First, The discovery capability was demonstrated by leaving five classes unlabled in the simulated data. However, I do not see such demonstration in the real dataset. Could this be done? Second, the proposed method was compared against the original DISCA model but would it be possible to also compare to other semi-supervised methods? These two simulations would strenghten the manuscript. Or I suggest that the authors include rationale on why they restrict to these comparisons.

Third, sensitivity analyses are lacking overall. Specifically, why 1% is chosen to enable semi-supervised learning? why not other proportions? Please explain the rationale or conduct sensitivity analyses for the parameters used in the models.

On the minor side, for Figure 2, have you verified the separation in t-SNE with UMAP or PCA plots to ensure that this is not an artifact?

Reviewer #2: This manuscript proposes a semi-supervised extension to the DISCA subtomogram clustering pipeline for cryo-electron tomography. The method introduces (i) a label-anchored/partially supervised GMM with labeled responsibilities fixed, (ii) multi-resolution PCA projection with majority voting to stabilize initialization, and (iii) an entropy-based confidence scoring and cosine-similarity label propagation scheme to refine pseudo-labels prior to iterative CNN retraining. The approach is evaluated on (a) simulated AITom datasets with 23 classes under varying SNR and tilt range, (b) a processed subset of the CZII CryoET Object Identification Challenge dataset with 3 annotated classes, and (c) PolNet simulated tomograms with 2 structures. Reported results show large improvements over DISCA (especially when DISCA does not recover the correct number of clusters) and improved feature separation in t-SNE visualizations.

Overall, the direction is compelling and potentially useful for structural discovery in cryo-ET where partial labels are common. However, several methodological clarifications and additional robustness evaluations are needed to support the strongest claims and to ensure reproducibility.

**********

**Figure resubmission:**

**Reproducibility:**

To enhance the reproducibility of your results, we recommend that authors of applicable studies deposit laboratory protocols in protocols.io, where a protocol can be assigned its own identifier (DOI) such that it can be cited independently in the future. Additionally, PLOS ONE offers an option to publish peer-reviewed clinical study protocols. Read more information on sharing protocols at https://plos.org/protocols?utm_medium=editorial-email&utm_source=authorletters&utm_campaign=protocols

# Author comment: Author response to Decision Letter 1

**DOI:** 10.1371/journal.pdig.0001619.r002

25 May 2026

Attachment Submitted filename: Response to Reviewers.pdf

# Peer review: Decision Letter 1

**DOI:** 10.1371/journal.pdig.0001619.r003

**Authors:** Ismini Lourentzou

13 Jul 2026

Semi-supervised clustering with knowledge-guided representation learning in cryo-electron tomography

PDIG-D-25-01241R1

Dear Dr. Xu,

We are pleased to inform you that your manuscript 'Semi-supervised clustering with knowledge-guided representation learning in cryo-electron tomography' has been provisionally accepted for publication in PLOS Digital Health.

Before your manuscript can be formally accepted you will need to complete some formatting changes, which you will receive in a follow-up email from a member of our team.

Please note that your manuscript will not be scheduled for publication until you have made the required changes, so a swift response is appreciated.

IMPORTANT: The editorial review process is now complete. PLOS will only permit corrections to spelling, formatting or significant scientific errors from this point onwards. Requests for major changes, or any which affect the scientific understanding of your work, will cause delays to the publication date of your manuscript.

If your institution or institutions have a press office, please notify them about your upcoming paper to help maximize its impact. If they'll be preparing press materials, please inform our press team as soon as possible -- no later than 48 hours after receiving the formal acceptance. Your manuscript will remain under strict press embargo until 2 pm Eastern Time on the date of publication. For more information, please contact digitalhealth@plos.org.

Thank you again for supporting Open Access publishing; we are looking forward to publishing your work in PLOS Digital Health.

Best regards,

Ismini Lourentzou

Section Editor

PLOS Digital Health

***********************************************************

**Additional Editor Comments (if provided):**

**Reviewer Comments (if any, and for reference):**

Reviewer's Responses to Questions

**Comments to the Author**

Reviewer #1: All comments have been addressed

Reviewer #2: All comments have been addressed

**********

publication criteria?

Reviewer #1: Yes

Reviewer #2: Yes

**********

3. Has the statistical analysis been performed appropriately and rigorously?

Reviewer #1: Yes

Reviewer #2: Yes

**********

4. Have the authors made all data underlying the findings in their manuscript fully available (please refer to the Data Availability Statement at the start of the manuscript PDF file)?

The PLOS Data policy

Reviewer #1: Yes

Reviewer #2: Yes

**********

5. Is the manuscript presented in an intelligible fashion and written in standard English?

Reviewer #1: Yes

Reviewer #2: Yes

**********

**Reviewer #1:** I have no additional comments.

**Reviewer #2:**  I thank the authors for their careful revision. In my view, the manuscript is now technically sound and suitable for publication. The proposed semi-supervised clustering framework is well motivated for cryo-electron tomography, where limited expert labels and large unlabeled datasets are common. The revised manuscript clearly describes the label-guided GMM initialization, PCA voting, confidence-based refinement, and iterative retraining pipeline, and the workflow figure is helpful for understanding the method.

The experimental results support the main conclusions. The method shows consistent improvements over DISCA and the fixed-k DISCA baseline on the AITom simulated datasets, including challenging low-SNR and limited-tilt settings. The CZII experiments further demonstrate applicability to experimental cryo-ET data, and the PolNet simulations provide additional evidence of robustness. The statistical and clustering evaluation metrics are appropriate for the study, including homogeneity, completeness, V-measure, and accuracy after optimal cluster-label matching.

I do not have remaining major concerns regarding the technical approach, presentation, research ethics, or competing interests. The manuscript is written clearly and is intelligible. I note that the authors state that data and code will be made publicly available through Hugging Face and GitHub upon acceptance; I consider this acceptable provided that the links are included and functional before publication.

Overall, I am satisfied that my concerns have been addressed and recommend acceptance.

**********

what does this mean?). If published, this will include your full peer review and any attached files.

**Do you want your identity to be public for this peer review?**  If you choose “no”, your identity will remain anonymous but your review may still be made public.

For information about this choice, including consent withdrawal, please see our Privacy Policy

Reviewer #1: No

Reviewer #2: **Yes:** Buse Demir

**********
